# Genome compaction underlies the molecular adaptation of bay cedar (*Suriana maritima*) to the extreme habitat on the tropical coral islands

**DOI:** 10.1016/j.pld.2025.01.002

**Published:** 2025-01-06

**Authors:** Miaomiao Shi, Ping Liang, Zhonglai Luo, Yu Zhang, Shiran Gu, Xiangping Wang, Xin Qian, Shuguang Jian, Kuaifei Xia, Shijin Li, Zhongtao Zhao, Tieyao Tu, Dianxiang Zhang

**Affiliations:** a State Key Laboratory of Plant Diversity and Specialty Crops / Guangdong Provincial Key Laboratory of Applied Botany / Key Laboratory of National Forestry and Grassland Administration on Plant Conservation and Utilization in Southern China, South China Botanical Garden, Chinese Academy of Sciences, Guangzhou 510650, China; b South China National Botanical Garden, Guangzhou 510650, China; c Department of Biological Sciences, Brock University, St. Catharines, Ontario, L2A 3S1, Canada; d School of Life Sciences and Medicine, Shandong University of Technology, Zibo 255000, China; e College of Life Sciences, Fujian Agriculture and Forestry University, Fuzhou 350002, China; f CAS Engineering Laboratory for Vegetation Ecosystem Restoration on Islands and Coastal Zones & Key Laboratory of Vegetation Restoration and Management of Degraded Ecosystems, South China Botanical Garden, Chinese Academy of Sciences, Guangzhou 510650, China

**Keywords:** Coral islands, Extreme environment, Gene loss, Genome reduction, Molecular adaptation, Transposable elements reduction

## Abstract

• The assembled genome of Bay cedar is 292.8 Mb, representing a small genome size within Fabales.
• The compact genome was likely caused by remarkable reduction of long terminal repeat retrotransposons and gene losses.
• The genes related to cold tolerance and pest/pathogen resistance were largely lost.
• Expanded, positively selected, or retained genes after WGD may drive Bay cedar's adaptation to tropical coral islands.
• Differentially expressed genes under salt and drought stresses were particularly identified in the abscisic acid pathway.

Tropical coral islands represent one of the extremely stressful ecosystems, characterized by high salinity, seasonal drought, heat, strong ultraviolet radiation, and infertile soil, which constraint species occurrence, limit plant growth and development, and reduce species richness comparing to tropical continental islands with mesophytic habitats (Li et al., 2024; Ren et al., 2017; Tu et al., 2022, 2024). Coupled with global climate changes, these adverse conditions have been being exacerbated, leading to extensive degradation of ecosystems throughout the tropical coral islands (Li et al., 2021). Native insular plant resources provide enormous potentials in island greening and ecological restoration, since they have colonized and become well adapted to the specialized habitat on tropical coral islands, evolving a series of functional traits and molecular strategies to accommodate the abiotic stresses. Thus, understanding the genomic make-up of these plants will help uncover molecular mechanisms underlying adaptation to tropical coral islands. However, contrary to the numerous genomic studies done for other extreme habitats, such as deserts (Hu et al., 2021; Ma et al., 2013), alpine regions (Zhang et al., 2023), intertidal habitats (Feng et al., 2021; Hu et al., 2020; Natarajan et al., 2021), and karst caves (Feng et al., 2020), molecular adaptation of plants on the tropical coral islands remains to be elucidated.

Surianaceae is a small family of Fabales consisting of five genera (*Cadellia*, *Guilfoylia*, *Recchia*, *Stylobasium*, and *Suriana*) and eight species, exclusively restricted in pantropics (Bello et al., 2012). Bay cedar (*Suriana maritima* L.) is the only species of the genus *Suriana*, growing as a perennial shrub or small tree. It occurs exclusively on sand dunes, shores, and rocky cracks, usually near the high tide marks (Liu et al., 2018) in coral islands and coasts of the tropic Pacific (Claxton et al., 2005). In China, it occurs exclusively as a dominant species on some tropical coral islands of Xisha Archipelago (or Paracel Islands) in South China Sea (Tu et al., 2022). Due to its excellent tolerance to salt, drought, heat, and infertile soil, bay cedar has been utilized in island greening and ecological restoration efforts in coral islands and coastal regions (Jian and Ren, 2017). This species has developed remarkable adaptations to thrive in the harsh environments of tropical coral islands, demonstrating an impressive level of resilience to adverse conditions (Zhou et al., 2023). The well-developed and deep underground roots of this species enhance its ability to stabilize sand and conserve water conservation effectively. Additionally, its small and thick leaves (Fig. S1), well-developed palisade tissue, and low stomatal density of leaves make it easy to reduce transpiration and maintain water in the body, enhancing the adaptive potentials on tropical coral islands. Thus, bay cedar represents an ideal model system for exploring the molecular mechanisms of adaptation to tropical coral islands. Here, we report a high-quality chromosome-level genome assembly of bay cedar (Fig. 1A), the first of such for the family Surianaceae, and further elucidate the genomic basis of its adaptation to tropical coral islands through comparative genomic and transcriptomic analyses.

**Fig. 1:**
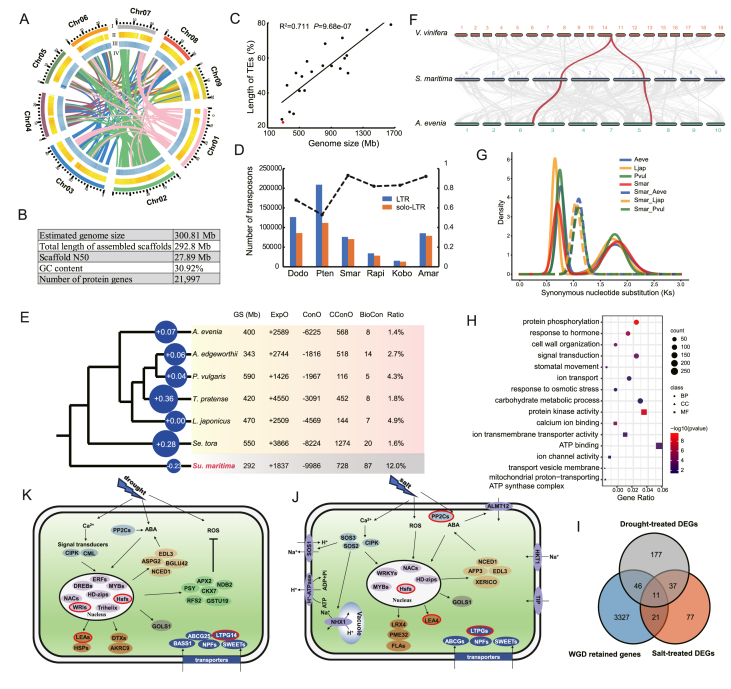
**A:** General characteristics of the bay cedar genome. From outside to inside: I, pseudochromosomes; II, gene density; III, repeat density; IV, gene collinearity. **B:** Statistics of the genome assembly. **C:** Scatter plots showing the relationship of genome size with transposable elements (TEs). **D:** Comparisons of bay cedar and five other species in LTRs (blue bars), solo-LTRs (orange bars) and ratio of solo-LTRs out of total LTR insertions (black dots). Dodo: *Dalbergia odorifera*; Pten: *Polygala tenuifolia*; Smar: *S. maritima*; Rapi: *Rhizophora apiculata*; Kobo: *Kandelia obovate*; Amar: *Avicennia marina*. **E:** Illustration of expansion and contraction of gene families on bay cedar and six Fagaceae species. Number of the average expanded genes of each branch are labeled in the circles; the size of the circles corresponds to the average expansion of each branch, where minus expansion indicates a net contraction. ExpO and ConO represent the number of expanded and contracted othorgroups compared to their common ancestors, respectively; CConO represents the number of contracted othorgroups in comparison with other five species; BioCon represents the number of contracted othorgroups associated with responses to biotic stresses; Ratio represents the ratio of BioCon/CConO. **F:** Diagram of syntenic blocks between genomic regions from *Vitis vinifera*, bay cedar and *Aeschnomene evenia*. Highlighted curves show a typical region in the *V. vinifera* genome can be traced to two regions each in bay cedar and *A. evenia*. **G:** Ks distributions of syntenic paralogous genes within the same species and orthologous genes between two species. Aeve: *A. evenia*; Ljap: *Lotus japonicus*; Pvul: *Phaseolus vulgaris*; Smar: *S. maritima.***H:** Gene Ontology enrichment among retained genes after the lineage-specific WGD. BP: Biological Process, CC: Cellular Component, and MF: Molecular Function. **I:** Venn diagram showing the relationships among retained genes from the lineage-specific WGD, DEGs under drought and salt treatments. **J and K:** The major salt (J) and drought (K) response regulatory pathways of bay cedar. Boxes with red borders indicate positively selection genes.

The bay cedar is diploid (2*n* = 18) (Liu et al., 2018) with an estimated haploid genome size of 300.81 Mb as determined based on k-mer distribution analysis of 24.72 Gb clean Illumina data (Fig. S2). The genome was initially assembled using 148× Nanopore long reads, yielding a total length of 292.80 Mb (Fig. 1B), covering 97.3% of the estimated genome size. The assembled genome comprised 172 contigs with a contig N50 length of 10.68 Mb. The longest scaffold was 42.93 Mb and scaffold N50 was 27.89 Mb. The GC content was 30.92%, representing a low level in the reported Fabales species. In addition, we generated a total of 41.52 Gb of clean reads using the Hi-C sequencing, anchoring and orienting 268.44 Mb (91.68% of total length) into 9 pseudochromosomes (Fig. S3 and Table S1). To evaluate the accuracy of the genome, we used BWA v.0.7.17 to align Illumina short reads to the assembly, and found that 99.24% of the Illumina PE reads mapped to the assembly. Conserved Core Eukaryotic Gene Mapping Approach (CEGMA) analysis showed that 452 of 458 core protein-coding genes (98.69%) were completely captured in the assembly. Bench-marking Universal Single-copy orthologs (BUSCO) analysis revealed that 94.03% of the core eudicotyledon genes were present (Table S2).

By using a combination of *de novo*-based, homology-based and RNA-Seq-based approaches, we obtained 21,997 protein-coding genes from the genome of bay cedar, representing 29.06% (in length of 85.09 Mb) of the genome (Fig. S4 and Table S3). On average, protein-coding genes are 3868 bp in length with 5.87 exons (Table S4). Among these genes, 21,366 (97.13%) were successfully annotated with putative functions (Table S5). In addition, we detected 71 microRNA (miRNA), 608 transfer RNA (tRNA), and 201 ribosomal RNA (rRNA) in the assembled genome (Table S6).

A total of 132 Mb (45.33%) of the bay cedar genome was identified as repetitive sequences (Table S7). Among these, transposable elements (TEs) accounts for 21.49% with long terminal repeat (LTR) retrotransposons as the largest fraction (18.56%). The *Copia* subfamily was the most abundant LTR type (9.08%), followed by *Gypsy* (6.15%). We found a significantly positive correlation between genome size and levels of repetitive elements, TEs, and LTR lengths across 21 Fabales species (Fig. 1C, Fig. S5; Table S8), suggesting that TE reduction likely contributes to the relatively small genome size of bay cedar. To investigate the mechanism of TE reduction, we estimated the rate of LTR removal using the ratio of solo-LTRs out of the total LTR insertions. The results showed that a higher ratio of solo-LTRs was observed in bay cedar as in the case the three other species living in stressful habitats (*Rhizophora apiculata, Kandelia obovata,* and *Avicennia marina*) (mean = 0.875) than the two species growing in non-extreme habitats (*Dalbergia odorifera* and *Polygala tenuifolia*) (mean = 0.605) (Fig. 1D). This result indicated that a higher rate of LTR removal rate from the homologous recombination-mediated deletion contributed at least as one factor for the lower LTR content in the genomes of species living in stressful conditions. Therefore, we hypothesize that an enhanced long-term selective pressures on the TE load likely led to the compact genome of bay cedar, primarily through LTR reduction, a pattern similar to that observed in mangrove species (Lyu et al., 2018).

To infer the phylogenetic position of bay cedar, a high-confidence phylogenetic tree was constructed using 1679 single-copy orthologous genes from seven Fabales species (*Aeschynomene evenia*, *Amphicarpaea edgeworthii*, *Lotus japonicus*, *Phaseolus vulgaris*, *Senna tora*, *Trifolium pratense*, and *S. maritima*) and two outgroups (*Arabidopsis thaliana* and *Prunus mume*) (Fig. S6). Based on the phylogeny and fossil calibration, bay cedar diverged from legume species around 78.19 million years ago (Mya) (95% HPD: 62.59–98.14 Mya) after the divergence from *P. mume* (Fig. S7). The divergence and other branching orders of the tree were consistent with the previous reports.

CAFÉ analysis identified 1837 expanded gene families in bay cedar compared to the common ancestor of Fabales. Among these, 11 gene families containing 158 genes significantly expanded (*P* < 0.01) (Fig. S7). Notably, we identified gene families previously associated with abiotic stress tolerance that expanded compared to the most recent common ancestor, including *HKT1*, *HSPs*, *APX1*, and *HAK5* (Table S9). Additionally, several transcription factor families, e.g., *MYB*, *NAC* and *WRKY*, exhibited expansion in the bay cedar genome, potentially enhancing regulatory responses to abiotic stresses. Furthermore, we identified 9986 contracted gene families in bay cedar, corresponding to the loss of 10,441 genes. Intriguingly, bay cedar exhibited the highest number of contracted gene families and the smallest number of average expanded genes (Fig. 1E). Comparing the number of genes in each orthogroup between bay cedar and species from the closely related family Fabaceae in mesophytic habitats revealed 728 orthogroups in bay cedar with fewer genes than any of the six Fabaceae species, of which 381 were completely lost. Among these contracted orthogroups, 12.0% were related to responses to biotic stresses, such as response to bacterium, fungi, herbivores, and pathogen resistance, e.g. leucine-rich repeat (LRR) proteins. Furthermore, there were several contracted orthogroups involving with response to cold, response to hormones (e.g., auxin, abscisic acid), cell wall organization and secondary metabolic processes (Table S10). These gene losses may suggest adaptive evolution in bay cedar on tropical coral islands, and may in turn restrict the distribution of this species to wider ecological niches but in the unique habitat.

Intra-genomic synteny analysis of bay cedar revealed 178 syntenic blocks with 2241 collinear gene pairs, representing 20.3% of its genes. Inter-genomic comparisons with *Vitis vinifera* showed a 2:1 syntenic depth pattern, with 27% *V. vinifera* genes corresponding to two syntenic blocks of bay cedar (Fig. 1F, Fig. S8), suggesting a whole-genome duplication (WGD) event after the well-known ancestral hexaploidization (WDT-γ). By estimating the distribution of the synonymous substitution divergence (*K*s), two distinct peaks were observed at *K*s ∼0.7 and 1.8 (Fig. 1G), further supporting a lineage-specific WGD (∼50.1 Mya) in bay cedar after its divergence from legume species. Following this WGD, 3405 duplicates (15.5% of total genes) were retained, with many enriched in GO terms related to ion transport, response to osmotic stress, calcium ion binding and superoxide metabolic process (Fig. 1H, Table S11). In the stress-related gene families identified and numerous transcription factor gene families, WGD-retained genes contributed 12.1%–72.7% (Tables S12 and S13). These preferential retentions pertained to adaptations in coral island habitats.

Comparative genomic analysis revealed 684 single-copy genes (18.8% of single-copy orthologs) under positive selection (Table S14). These genes are associated with some basic life processes, including reproductive system, flower and shoot developments, response to oxidative stress, ion transport, heat shock protein binding, and osmosensor activity (Table S15; Fig. S9), contributing to reactive oxygen species (ROS) removal (e.g., *PER1*, *APX6*, *F3H*) and cellular environmental homeostasis (e.g., *BASS3*, *NPF5.1*, *LITP2*) in bay cedar under abiotic stresses.

To further examine the genome-wide responses to salt and drought stresses in bay cedar, we conducted experimental manipulations and performed transcriptome sequencings on root samples. We identified 271 and 146 differentially expressed genes (DEGs), respectively, under drought and salt stresses comparing with control samples (Fig. 1I). Among them, 261 were significantly upregulated (Tables S16 and S17), including numerous genes of signal transduction (e.g., *CDPK*, *PP2C*), protectant proteins (e.g., *LEA*s), and transcription factors (e.g., *DREB*, *ERF*, *MYB*, and *HSF*) (Fig. 1J and K). Notably, there were over 15 genes that were directly involved in the abscisic acid (ABA) pathways, including *NCED1*, *CYP707A4*, *EDL3*, *BGLU42*, *XERICO*, *PP2C*s and *DREB*s, which highlighted the significance of ABA relevant genes in coping with drought and salt stresses. Also, we found numerous transporter genes upregulated among DEGs, including *ABCG*s, *NPF*s, *LTPG*s and *SWEET*s (Fig. 1J and K). These transporters help move metabolites and nutrients across membrane and are crucial for cellular homeostasis and stress responses. In addition, there were 48 shared DEGs between the two abiotic stresses with 38 upregulated. Among these, 11 were retained from the lineage-specific WGD event (Fig. 1I) with two as a duplicated gene pair (encoding *PP2C37*), while the other nine genes representing only one copy of the duplicate genes. The latter indicates that a number of duplicated paralogous genes from WGD in bay cedar have evolved novel functions during its adaptation to coral islands.

In summary, we present here a high-quality chromosome-level reference genome of bay cedar, the first in Surianaceae. The assembled genome size (∼292.80 Mb) represents a very compact genome comparing to its legume relatives, probably due to TE reductions and massive gene losses caused by long-term selection pressures. The genes related to cold tolerance and pest/pathogen resistance were largely lost. The expanded gene families, positively selected genes, and retained genes from the lineage-specific WGD probably reflect the adaptation of bay cedar to salinity and seasonal drought on coral islands. From transcriptomic analysis, we deduced that ABA pathway and the maintenance of cellular homeostasis are crucial adaptive processes in bay cedar. The genomic resources reported here offer valuable information that will facilitate the evolutionary and ecological and genomics studies in bay cedar and related species. Our study highlights the directions of the molecular evolution of the species and the expectations of future adaptations in response to climatic and environmental changes in different lineages. Our study also provides insights into how plants adapt to harsh and extreme environments on tropical coral islands, and such knowledge is expected to help with our future effort for island greening and ecological restoration.

## CRediT authorship contribution statement

**Miaomiao Shi:** Writing – original draft, Visualization, Methodology, Formal analysis, Data curation. **Ping Liang:** Methodology, Data curation. **Zhonglai Luo:** Methodology, Data curation. **Yu Zhang:** Methodology, Formal analysis. **Shiran Gu:** Formal analysis. **Xiangping Wang:** Methodology, Investigation. **Xin Qian:** Methodology, Investigation. **Shuguang Jian:** Methodology. **Kuaifei Xia:** Methodology. **Shijin Li:** Writing – review & editing, Methodology. **Zhongtao Zhao:** Writing – review & editing, Methodology, Formal analysis, Data curation. **Tieyao Tu:** Writing – review & editing, Conceptualization. **Dianxiang Zhang:** Writing – review & editing, Conceptualization.

## Data availability

The raw Illumina genome sequencing reads have been deposited in the National Center for Biotechnology Information (NCBI) under BioProject ID PRJNA1079739. The raw Nanopore long reads and transcriptome data have been submitted to NCBI under Bioproject ID PRJNA1095649. All the supplementary materials that support the findings of this study are available in Appendix B.

## Declaration of competing interest

The authors declare that they have no known competing financial interests or personal relationships that could have appeared to influence the work reported in this paper.
