# Supplementary material for: Genome compaction underlies the molecular adaptation of bay cedar (Suriana maritima) to the extreme habitat on the tropical coral islands
Source: Plant Divers. 2025 Jan 6;47(2):337–40. doi: 10.1016/j.pld.2025.01.002 (PMC11962907; doi:10.1016/j.pld.2025.01.002)
Supplement: Multimedia component 1 [file mmc1.docx]

**Genome compaction underlies the molecular adaptation of Bay cedar to the extreme habitat on the tropical coral islands**

Miaomiao Shi, Ping Liang, Zhonglai Luo, Yu Zhang, Shiran Gu, Xiangping Wang, Xin Qian, Shuguang Jian, Kuaifei Xia, Shijin Li, Zhongtao Zhao*, Tieyao Tu*, Dianxiang Zhang

**SUPPORTING DATA**


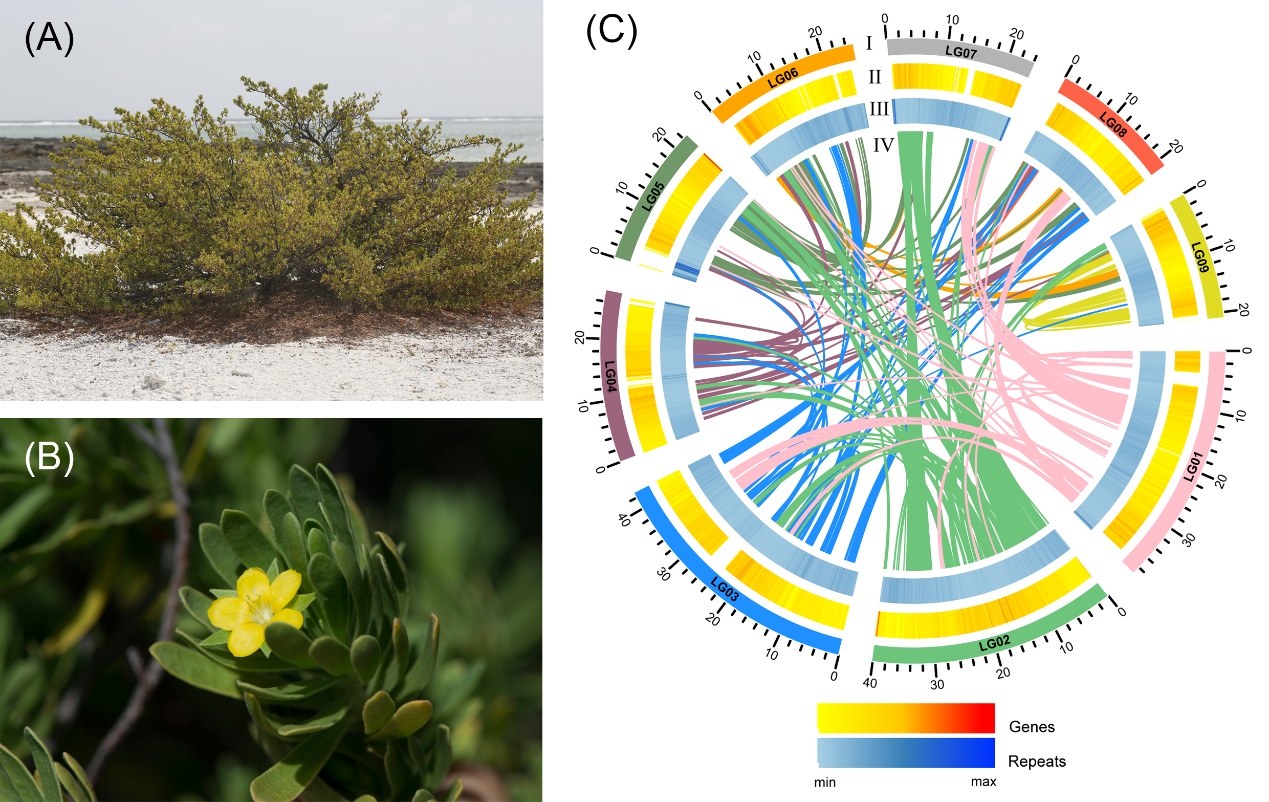

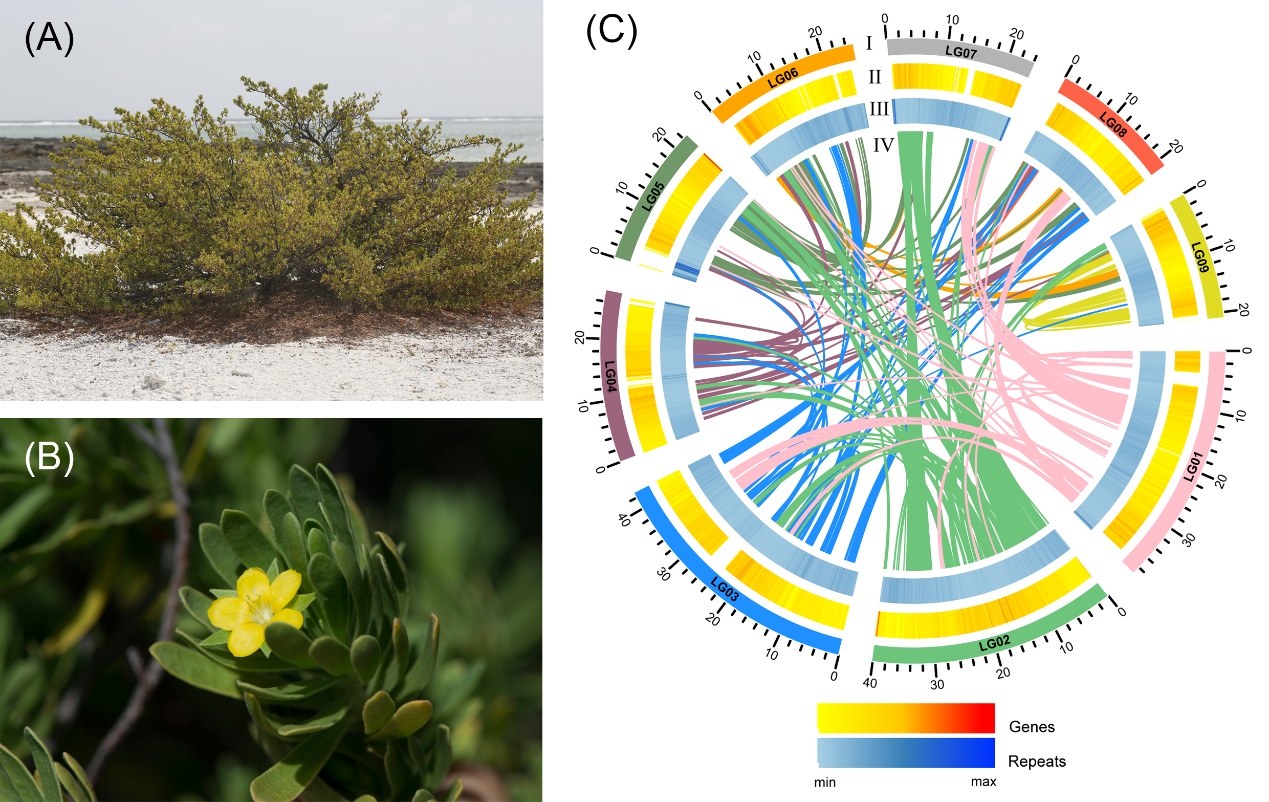


**Fig. S1.** Photographs of Bay cedar showing the habitats on the Paracel Islands (A) and its leaves and flower.

**
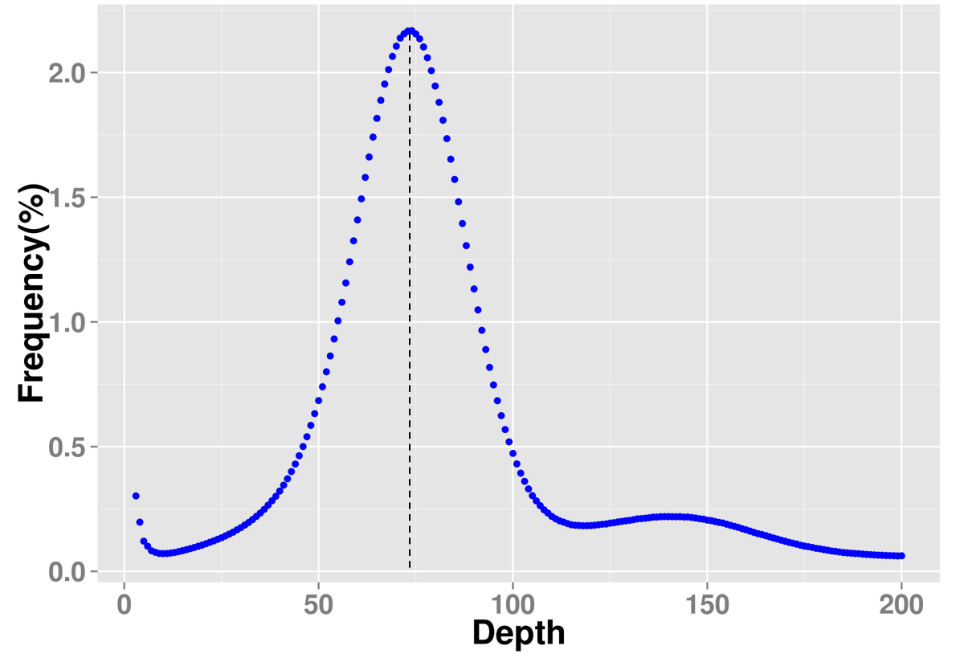
**

**Fig. S2**. K-mer frequency distribution curve (k = 17) based on Illumina short reads of *Suriana maritima*.


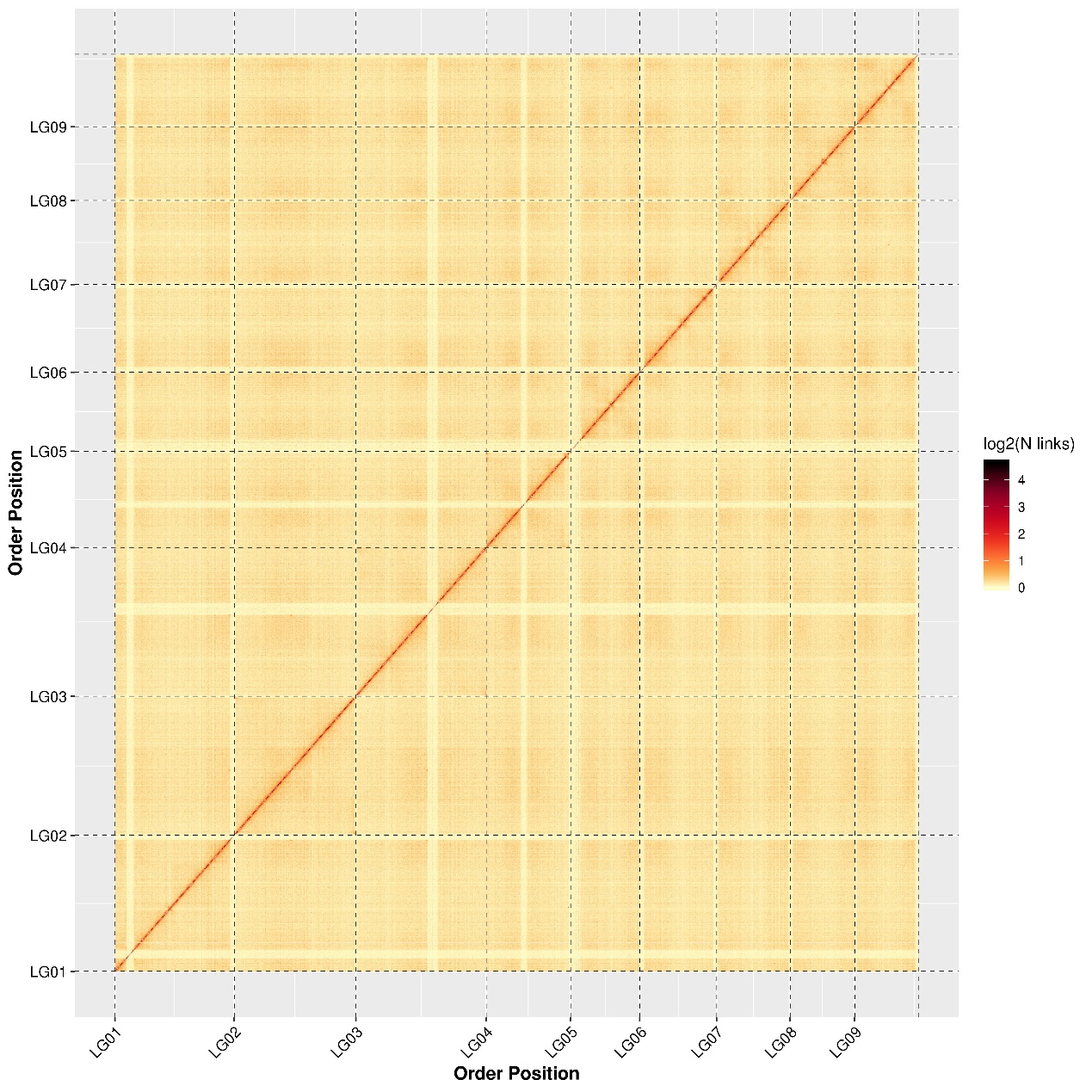


**Fig. S3**. Hi-C contact data mapped to the genome of *Suriana maritima*. The heat map represents the normalized contact matrix. The strongest and weakest contact are shown in red and yellow, respectively.


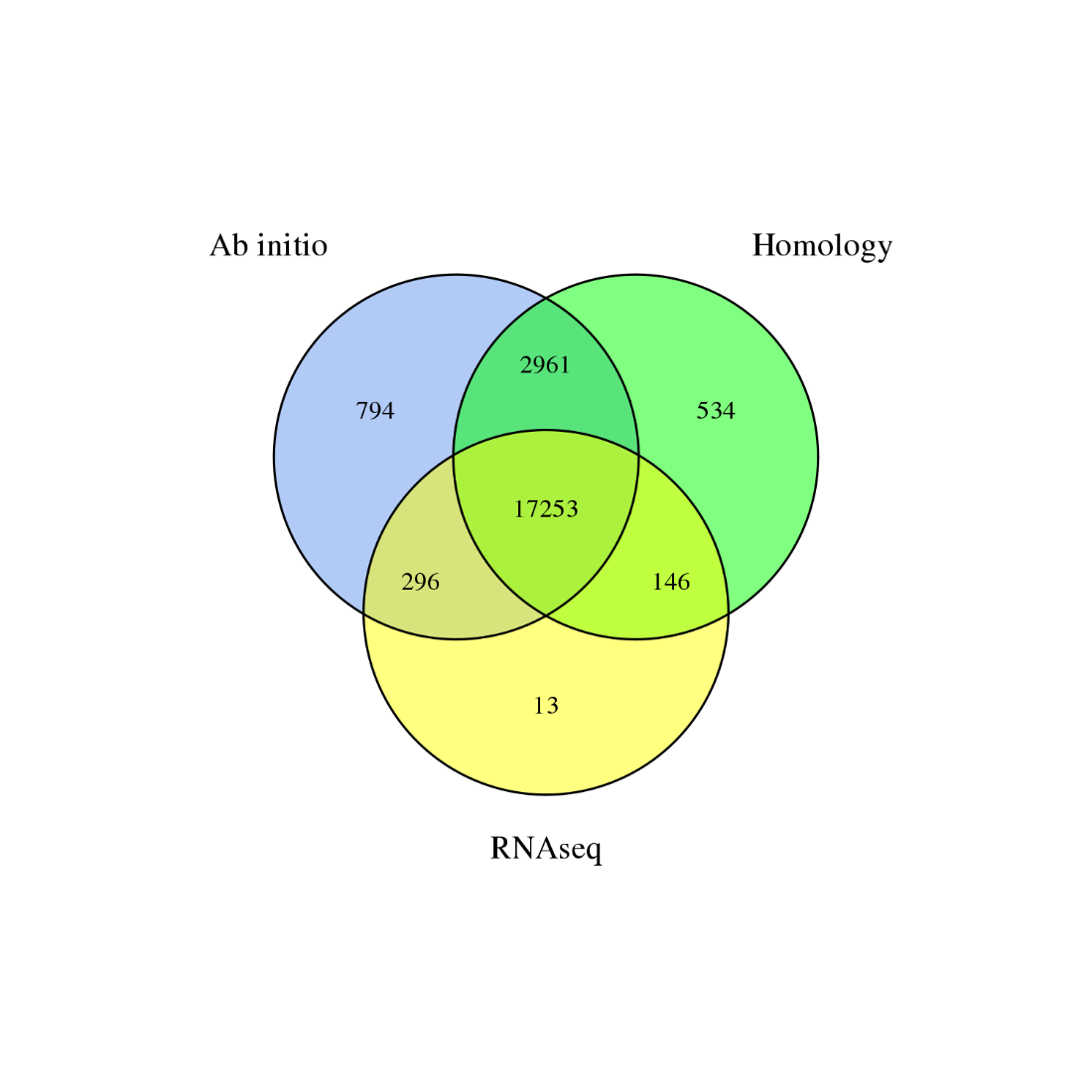


*De novo*

Homology

RNA-seq

Integration 21,997

**Fig. S4**. Number of genes predicted with *de novo*, homology and RNA-seq.


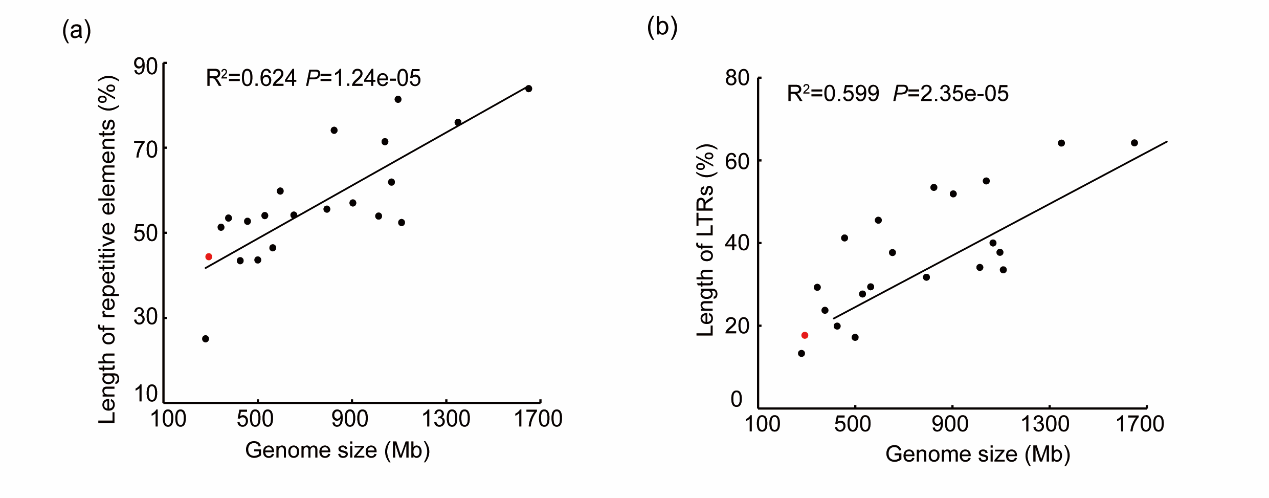


**Fig. S5.** Scatter plots showing the relationships of genome size with repetitive elements (A), and long terminal repeat retrotransposons (LTR) (B) within 21 Fabales species. The red points indicate the values of Bay cedar.


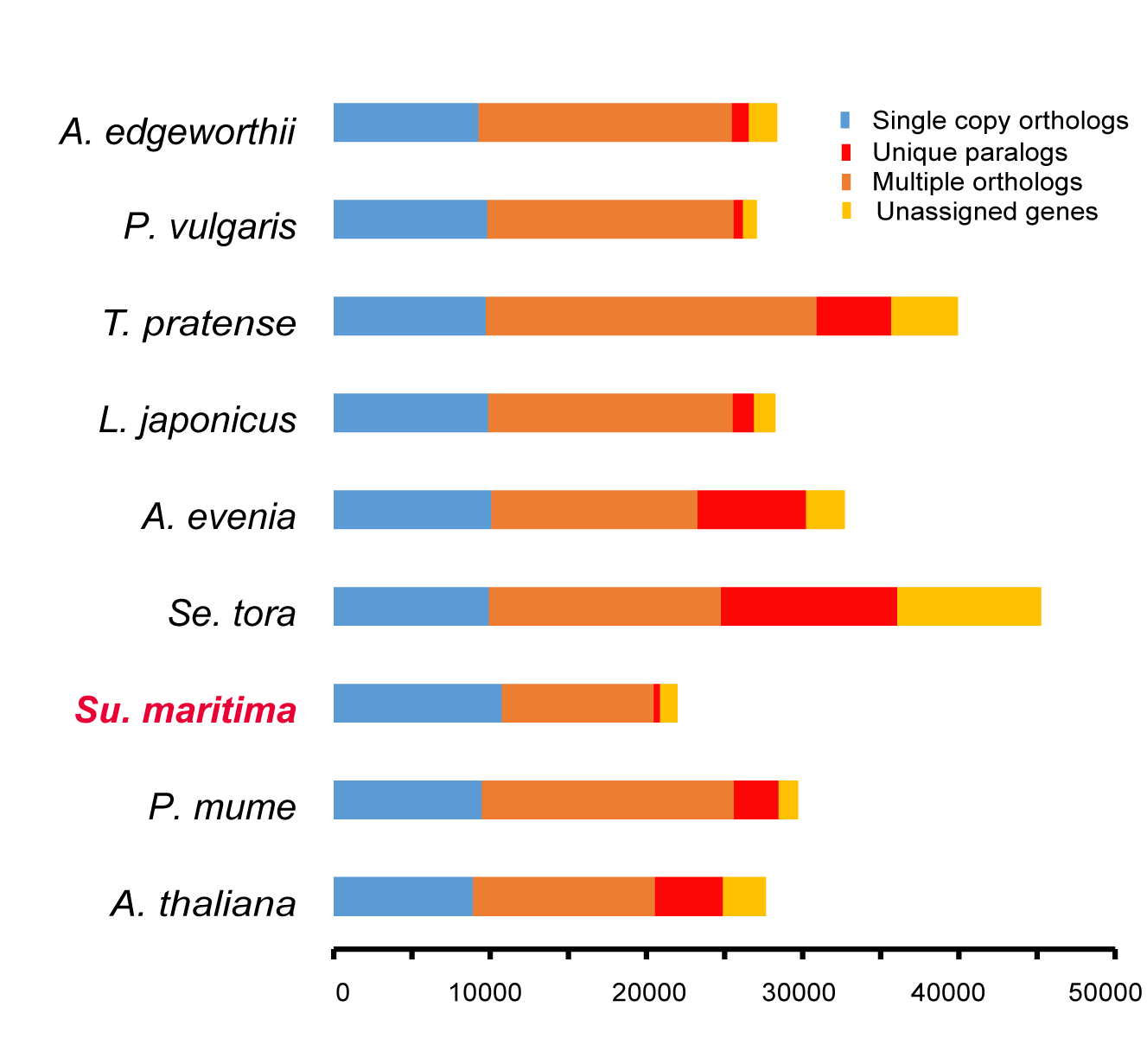


**Fig. S6.** Clusters of orthologous and paralogous gene families in Bay cedar and other comparative species.


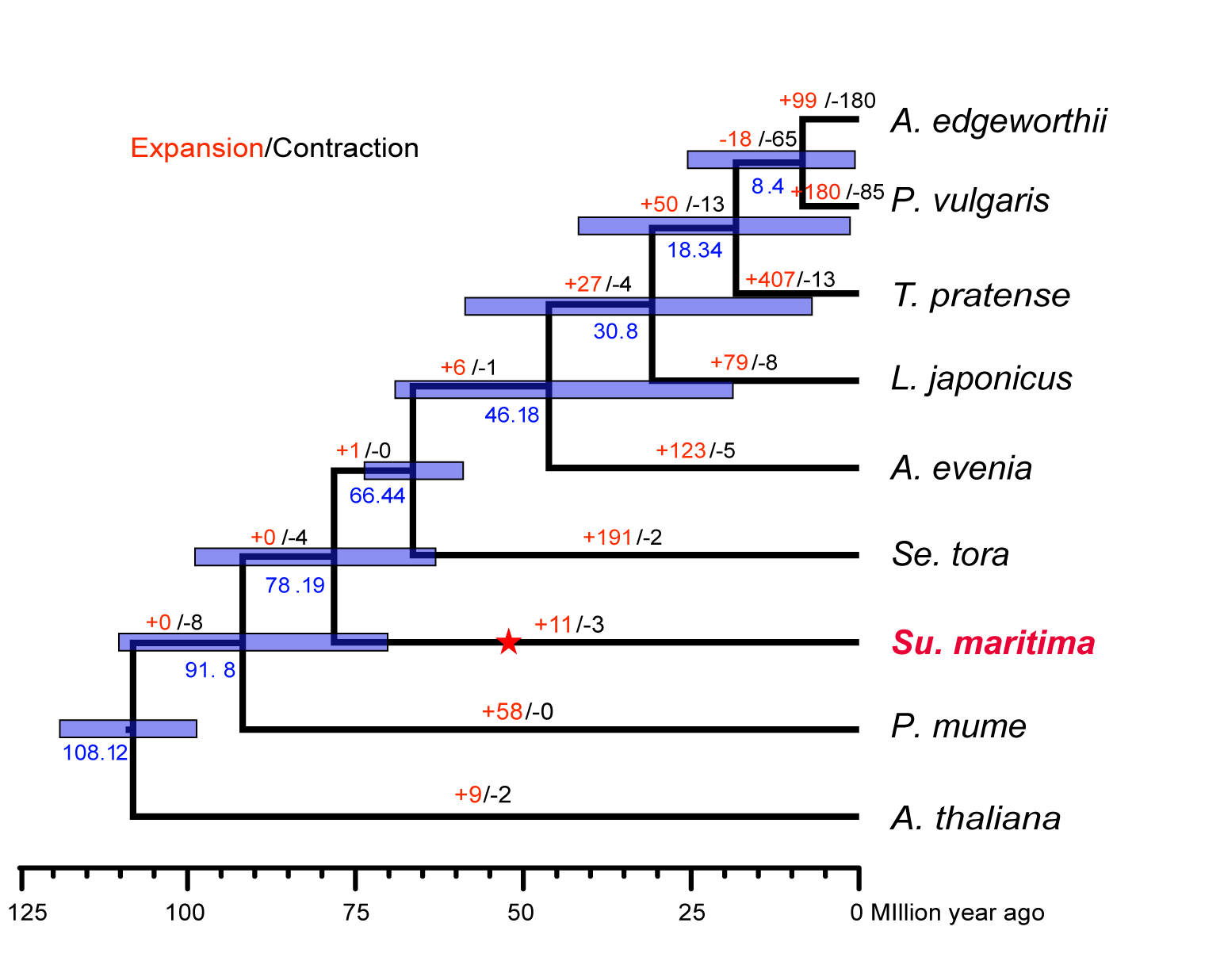


**Fig. S7.** Phylogenetic relationship between Bay cedar and other representative plant species. The numbers in blue below each node shows the estimated divergent time of each node (Mys) and the bars represent 95% confidence intervals for the time of divergence. Red and black numbers on each branch of the tree indicate the significantly expanded and contracted gene families, respectively. The asterisks represent the estimated date of WGD events.


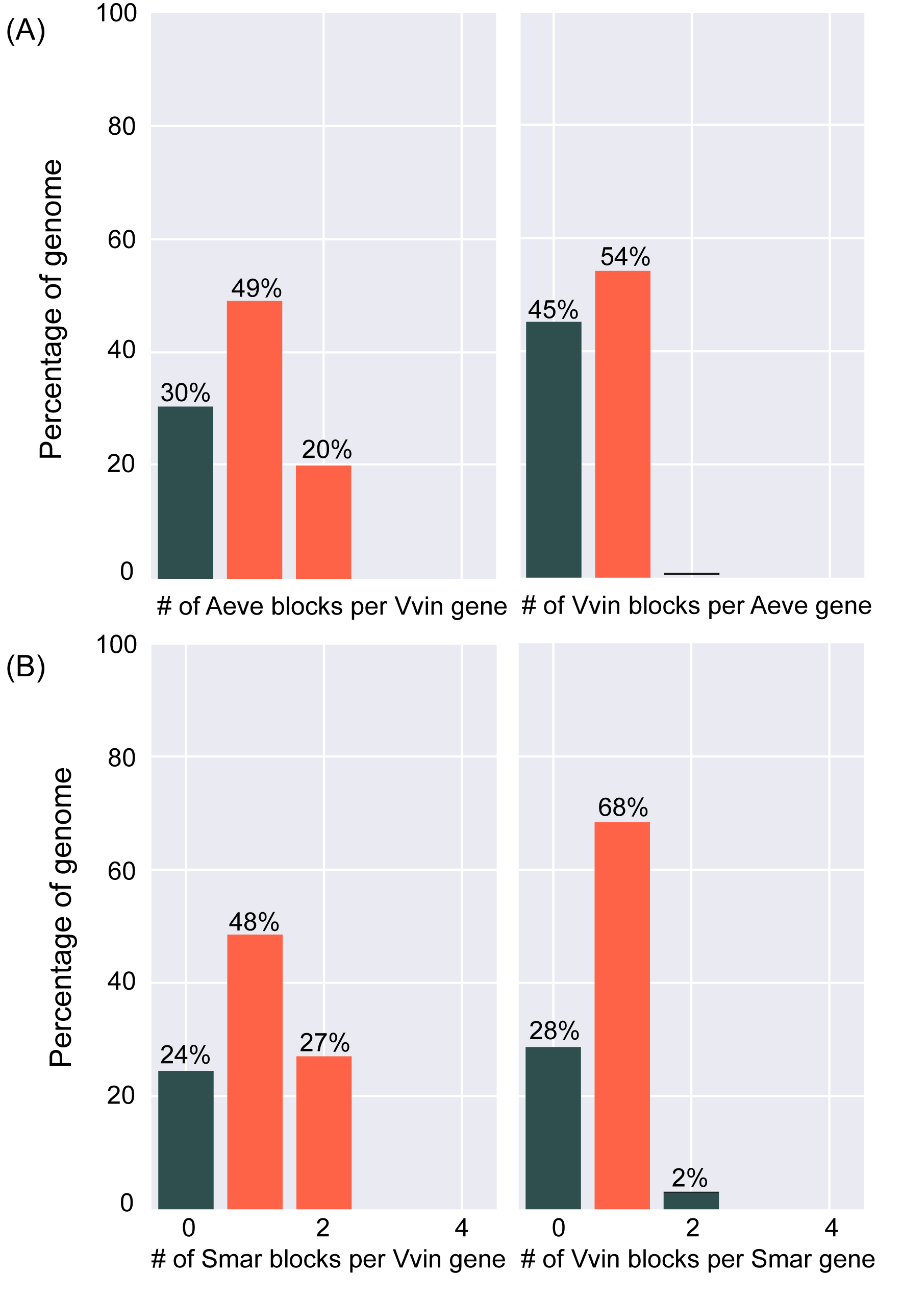


**Fig. S8.** Syntenic depths (A) between *Aeschnomene evenia* (Aeve) and *Vitis vinifera* (Vvin) (2:1), and (B) between *S. maritima* (Smar) and *V. vinifera* (2:1).


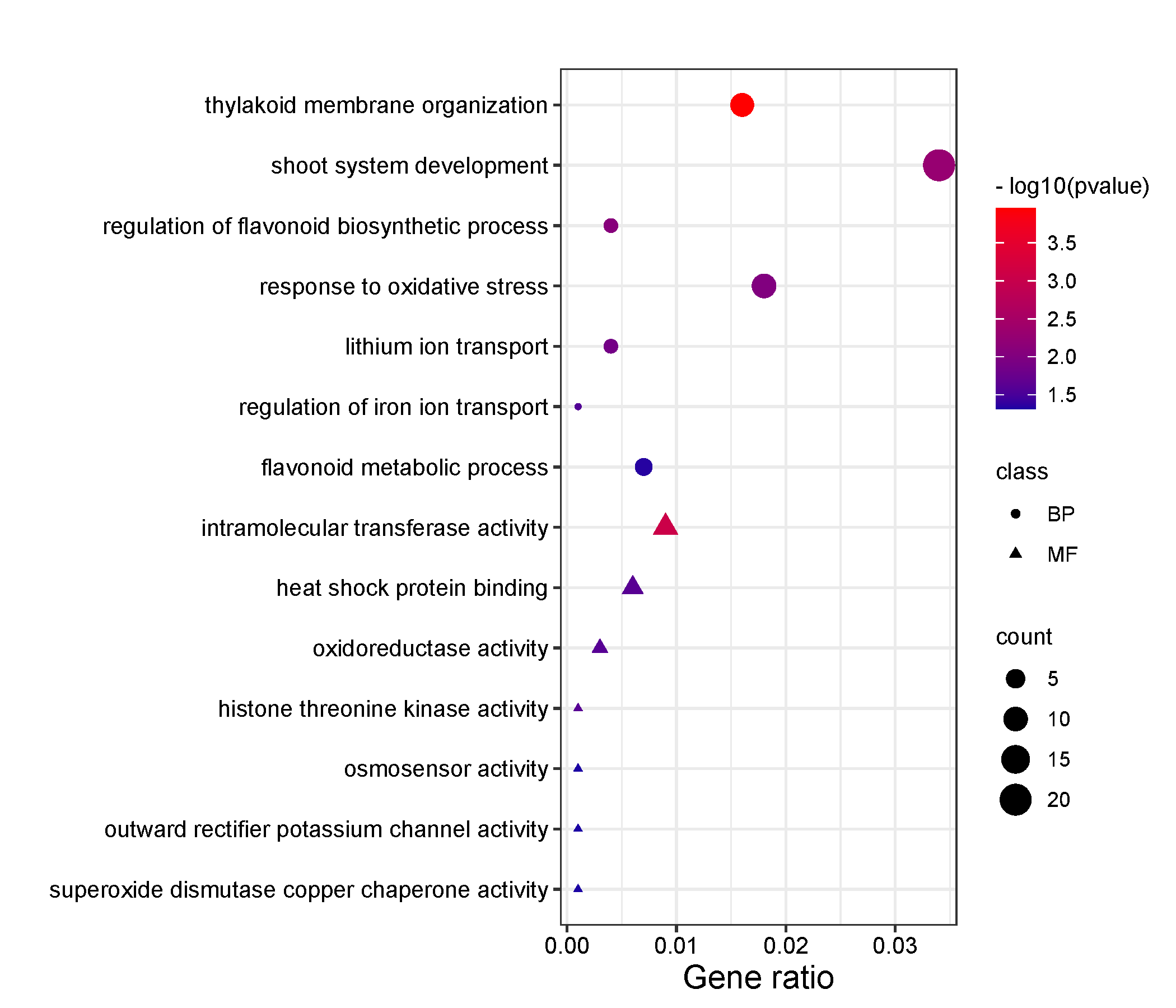


**Fig. S9.** Gene Ontology enrichment for single-copy genes under positive selection in *S. maritima*.

**Table S1.** Assembly statistics based on Hi-C data.

| Group | Cluster Number | Cluster Length (bp) | Order Number | Order Length (bp) |
| --- | --- | --- | --- | --- |
| LG01 | 12 | 39,642,577 | 10 | 39,329,639 |
| LG02 | 10 | 40,624,356 | 6 | 40,185,856 |
| LG03 | 10 | 43,286,924 | 7 | 42,932,458 |
| LG04 | 9 | 28,015,527 | 7 | 27,887,503 |
| LG05 | 20 | 23,688,909 | 13 | 22,893,697 |
| LG06 | 8 | 25,512,521 | 7 | 25,304,880 |
| LG07 | 11 | 24,697,189 | 8 | 24,264,347 |
| LG08 | 5 | 21,739,461 | 4 | 21,375,235 |
| LG09 | 6 | 21,233,778 | 4 | 20,916,557 |
| Total | 91 | 268,441,242 | 66 | 265,090,172 |
| Ratio % | 38.72 | 91.68 | 72.53 | 98.75 |

**Table S2.** Genome assembly completeness evaluation by BUSCO.

| Type | Number | Percentage |
| --- | --- | --- |
| Complete BUSCOs | 1,354 | 94.03% |
| Complete and single-copy BUSCOs | 1,311 | 91.04% |
| Complete and duplicated BUSCOs | 43 | 2.99% |
| Fragmented BUSCOs | 21 | 1.53% |
| Missing BUSCOs | 64 | 4.44% |
| Total BUSCO groups searched | 1,440 | 100% |

**Table S3.** Summary of predicted protein-coding genes using three methods in the Suriana maritima genome.

| Method | Software | Species | Gene number |
| --- | --- | --- | --- |
| *de novo* | Genscan |  | 19,925 |
|  | Augustus |  | 20,498 |
|  | GlimmerHMM |  | 32,195 |
|  | GeneID |  | 32,695 |
|  | SNAP |  | 39,278 |
| Homology-based | GeMoMa | *Arabidopsis thaliana* | 19,352 |
|  |  | *Arachis hypogaea* | 20,945 |
|  |  | *Glycine max* | 22,733 |
|  |  | *Oryza sativa* | 19,191 |
| RNA-seq | TransDecoder |  | 65,390 |
|  | GeneMarkS-T |  | 39,548 |
|  | PASA |  | 50,880 |
| Integration | EVM |  | 21,997 |

**Table S4.** Statistics of predicted protein-coding genes in the *Suriana maritima* genome.

| Feature | Value |
| --- | --- |
| Number of genes | 21,997 |
| Total length of genes (Mb) | 85.09 |
| Average length of genes (bp) | 3,868.20 |
| Total length of exons (Mb) | 38.04 |
| Average length of exon per gene (bp) | 1,729.52 |
| Number of exons | 129,046 |
| Average number of exons per gene | 5.87 |
| Total length of CDS (Mb) | 28.14 |
| Average length of CDS per gene (bp) | 1,279.42 |
| Number of CDS | 123,983 |
| Average number of CDS per gene | 5.64 |
| Total length of introns (Mb) | 47.04 |
| Average length of intron per gene (bp) | 2,138.68 |
| Number of introns | 107,049 |
| Average number of introns per gene | 4.87 |

**Table S5.** Summary of protein-coding gene annotation of *Suriana maritima*.

| Database | Number | Percentage (%) |
| --- | --- | --- |
| GO | 13,186 | 59.94 |
| KEGG | 8,167 | 37.13 |
| KOG | 12,050 | 54.78 |
| TrEMBL | 21,340 | 97.01 |
| NR | 21,355 | 97.08 |
| All | 21,366 | 97.13 |

**Table S6.** Statistics of the noncoding RNA in the *Suriana maritima* genome.

| RNA classification | Number | Family |
| --- | --- | --- |
| miRNA | 71 | 20 |
| rRNA | 201 | 4 |
| tRNA | 608 | 24 |

**Table S7.** Statistics of the repeat sequences in the genomes of *Suriana maritima* and comparative species**.**

| Type | *Dalbergia odorifera* | *Polygala tenuifolia* | ***Suriana maritima*** | *Rhizophora apiculata* | *Kandelia obovata* | *Avicennia marina* |
| --- | --- | --- | --- | --- | --- | --- |
| DNA | 2.90% | 6.44% | 1.94% | 1.11% | 0.16% | 3.22% |
| LINE | 0.99% | 0.84% | 0.99% | 0.90% | 0.36% | 0.66% |
| SINE | 0 | 0 | 0 | 0.03% | 0 | 0 |
| LTR | 23.12% | 30.94% | 18.56% | 17.16% | 11.79% | 22.82% |
| LTR-Copia | 4.94% | 3.63% | 9.08% | 8.92% | 5.68% | 9.98% |
| LTR-Gypsy | 13.81% | 24.67% | 6.15% | 6.58% | 5.21% | 12.12% |
| LTR-ERVK | 0.00% | 0.00% | 0.00% | 0.00% | 0.00% | 0.02% |
| LTR-Caulimovirus | 0.33% | 0.53% | 1.18% | 1.38% | 0.25% | 0.56% |
| LTR-Ngaro | 0.00% | 0.00% | 0.00% | 0.00% | 0.00% | 0.09% |
| LTR-Pao | 0.06% | 0.00% | 0.00% | 0.00% | 0.00% | 0.00% |
| LTR-unknown | 3.98% | 2.11% | 2.15% | 0.28% | 0.65% | 0.05% |
| Low complexity | 0 | 0 | 0.01% | 0 | 0 | 0 |
| Satellite | 0 | 0 | 0.19% | 0 | 0 | 0 |
| Simple repeat | 0.02% | 0.02% | 0.05% | 0.28% | 0.15% | 0 |
| unknown | 33.32% | 26.13% | 23.45% | 11.21% | 4.26% | 23.85% |
| Total | 60.86% | 64.58% | 45.33% | 30.93% | 16.72% | 50.61% |

**Table S8.** A comparison of *S. maritima* with 20 legume species in terms of genome size, and the proportions of repetitive elements, transposable elements (TEs) and long terminal repeat retrotransposons (LTRs).

| Species | Size of genome | Repetitive elements (%) | TE(%) | LTR (%) | Reference |
| --- | --- | --- | --- | --- | --- |
| *Ammopiptanthus nanus* | 823.74 | 74.08 | 68.1 | 53.43 | Gao et al., 2018 |
| *Amphicarpaea edgeworthii* | 343.78 | 51.28 | 44.34 | 29.32 | Song et al., 2021 |
| *Medicago sativa* | 793.2 | 55.55 | 55.55 | 31.75 | Li et al., 2020 |
| *Aeschynomene evenia* | 376 | 53.47 | 29.16 | 23.75 | Quibe et al., 2021 |
| *Astragalus sinicus* | 595.52 | 59.84 | 58 | 45.52 | Chang et al., 2021 |
| *Dalbergia odorifera* | 653.45 | 54.17 | 52.13 | 37.7 | Hong et al., 2020 |
| *Entada phaseoloides* | 456.18 | 52.7 | 51.38 | 41.24 | Lin et al., 2022 |
| *Glycine max* | 1013.2 | 53.9 | 53.9 | 34.1 | Kim et al., 2021 |
| *Macrotyloma uniflorum* | 279.12 | 25.04 | 24.91 | 13.31 | Shirasawa et al., 2021 |
| *Sindora glabra* | 1110 | 52.4 | 51.32 | 33.53 | Yu et al., 2022 |
| *Arachis duranensis* | 1067.49 | 61.89 | 59.77 | 40 | Bertioli et al., 2016 |
| *Melilotus albus* | 1040 | 71.42 | 71.42 | 54.99 | Wu et al., 2021 |
| *Cicer arietinum* | 530.27 | 54.03 | 49.39 | 27.66 | Parween et al., 2015 |
| *Medicago truncatula* | 500 | 43.61 | 41.21 | 17.21 | Young et al., 2011 |
| *Lablab purpureus* | 426.2 | 43.4 | 28.1 | 19.9 | Njaci et al., 2023 |
| *Arachis ipaensis* | 1349.51 | 75.97 | 75.97 | 64.15 | Bertioli et al., 2016 |
| *Medicago ruthenica* | 904.13 | 57 | 55.4 | 51.9 | Wang et al., 2021 |
| *Vicia sativa* | 1650 | 83.92 | 79 | 64.2 | Xi et al., 2022 |
| *Trifolium repens* | 1096 | 81.37 | 61.37 | 37.75 | Wang et al., 2023 |
| *Vigna unguiculata* | 564 | 46.47 | 41.4 | 29.39 | Liang et al., 2022 |
| *Suriana maritima* | 292.8 | 37.09 | 28.95 | 18.35 | this study |

**Table S9.** Expanded gene families related to abiotic stresses and their functions.

| Gene family | No. of genes | Function |
| --- | --- | --- |
| AHK5 | 8 | Functions as a histidine kinase and transmits the stress signal to a downstream MAPK cascade |
| ALMT12 | 11 | Aluminum-activated malate transporter 12, involved in dark-, CO2-, abscisic acid- and water-deficient-induced stomatal closure. |
| APX1 | 7 | L-ascorbate peroxidase 1, plays a key role in hydrogen peroxide removal |
| ANN1 | 10 | Annexin D1, has a peroxidase activity, may act in counteracting oxidative stress |
| AKT1 | 8 | Potassium channel AKT1 |
| DHAR | 3 | Glutathione S-transferase, scavenging of ROS under oxidative stresses |
| GPX | 7 | Glutathione peroxidase, may protect cells and enzymes from oxidative damage |
| HKT1 | 3 | Sodium transporter |
| HSP | 16 | Heat shock protein |
| LEA | 4 | Late embryogenesis abundant protein,involved in the adaptive response of vascular plants to withstand water deficit |
| MSD | 3 | Superoxide dismutase, destroys superoxide anion radicals |
| MDAR | 5 | Monodehydroascorbate reductase |
| NHX | 7 | Sodium/hydrogen exchanger |
| CIPK | 23 | CBL-interacting serine/threonine-protein kinase |
| CML | 25 | Calmodulin-like protein |
| CDPK | 32 | Calcium-dependent protein kinase |
| CBL | 10 | Calcineurin B-like proterin |
| MKK | 7 | Mitogen-activated protein kinase kinase |
| HAK5 | 21 | Potassium transporter |
| NPF | 51 | Dual affinity nitrate transporter |
| TIP | 11 | Aquaporin |
| PIP | 13 | Aquaporin |
| SAP | 9 | Zinc finger A20 and AN1 domain-containing stress-associated protein, involved in environmental stress response |
| SLAC | 6 | Guard cell S-type anion channel, involved in maintenance of anion homeostasis |
| PKS | 19 | protein kinase |
| H^+^-ATPase | 13 | Plasma membrane, generates a proton gradient and provide the driving force for active transport of ions |
| 14-3-3 | 11 | A molecular switch in salt stress tolerance, decodes a salt-induced calcium signal to enhance salt tolerance |

**Table S10. Contracted gene families of *Suriana maritia* in comparison with six Fabaceae species associated with responses to biotic and abiotic stresses.**

| Family ID | Encoded protein | function | *Suriana maritima* | *Trifolium pratense* | *Lotus japonicus* | *Senna tora* | *Phaseolus vulgaris* | *Aeschnomene evenia* | *Amphicarpaea edgeworthii* |
| --- | --- | --- | --- | --- | --- | --- | --- | --- | --- |
| OG0000094 | Salicylate/benzoate carboxyl methyltransferase | defense to herbivore | 5 | 15 | 20 | 10 | 13 | 7 | 6 |
| OG0000179 | Heavy metal-associated isoprenylated plant protein HIPP39 | resistance to stress | 4 | 10 | 8 | 5 | 13 | 10 | 5 |
| OG0000064 | Linoleate 9S-lipoxygenase 1 | limit pathogen infection | 3 | 18 | 14 | 8 | 21 | 11 | 6 |
| OG0000370 | Aquaporin PIP1.1 | response to water deprivation | 3 | 5 | 5 | 4 | 5 | 5 | 5 |
| OG0000512 | WRKY33 | defense response to fungus | 3 | 4 | 6 | 4 | 4 | 4 | 4 |
| OG0000010 | LRR receptor-like kinase resistance protein | plant immunity | 2 | 36 | 15 | 25 | 46 | 10 | 3 |
| OG0000050 | LRR receptor-like kinase family protein | plant immunity | 2 | 20 | 9 | 24 | 8 | 12 | 13 |
| OG0000082 | Cytochrome P450 81D11 | defense to aphids | 2 | 12 | 6 | 9 | 14 | 15 | 3 |
| OG0000218 | (E)-beta-ocimene synthase | defense to herbivore | 2 | 3 | 9 | 3 | 11 | 12 | 5 |
| OG0000373 | Wound-responsive family protein | response to wound | 2 | 9 | 3 | 3 | 5 | 4 | 7 |
| OG0000388 | Subtilisin-like protease SBT4.4 | plant defense (plant-pathogen) | 2 | 3 | 11 | 5 | 3 | 6 | 3 |
| OG0000545 | E3 ubiquitin-protein ligase RGLG2 | response to drought stress | 2 | 4 | 3 | 4 | 4 | 5 | 4 |
| OG0000628 | Ethylene-responsive transcription factor RAP2-1 | response to cold, water deprivation | 2 | 3 | 3 | 4 | 4 | 3 | 5 |
| OG0000878 | AP2-like ethylene-responsive transcription factor | response to environmental stimuli | 2 | 4 | 3 | 3 | 4 | 4 | 4 |
| OG0000944 | calcium-dependent protein kinase SK5 | plant immunity | 2 | 4 | 3 | 3 | 3 | 3 | 3 |
| OG0001056 | Glycine-rich RNA-binding protein 3, mitochondrial RBG3 | response to cold | 2 | 3 | 3 | 3 | 3 | 3 | 3 |
| OG0001092 | RING-H2 finger protein ATL80 | response to cold, defense response | 2 | 3 | 3 | 3 | 3 | 4 | 3 |
| OG0000083 | LRR Leucine-rich repeat domain containing protein | plant immunity | 1 | 25 | 13 | 11 | 11 | 6 | 3 |
| OG0000144 | Probable disease resistance protein | defense response to fungus | 1 | 21 | 7 | 3 | 8 | 4 | 6 |
| OG0000222 | S-Domain receptor like kinase-1 | plant immunity | 1 | 12 | 5 | 4 | 13 | 6 | 7 |
| OG0000312 | Ethylene-responsive transcription factor 1B | defense against fungi | 1 | 8 | 8 | 6 | 8 | 6 | 2 |
| OG0000347 | 12-oxophytodienoate reductase 1 OPR1 | defense against stress | 1 | 8 | 4 | 2 | 8 | 4 | 4 |
| OG0000444 | Amine oxidase [copper-containing] alpha 3 | response to water deprivation, wounding | 1 | 6 | 4 | 5 | 6 | 6 | 3 |
| OG0000463 | Flavonoid 3'-monooxygenase CYP75B1 | response to auxin | 1 | 9 | 5 | 8 | 4 | 4 | 3 |
| OG0000469 | Aldo/keto reductase family oxidoreductase AKR | defense against abiotic stresses | 1 | 5 | 14 | 3 | 6 | 2 | 2 |
| OG0000562 | S-Domain receptor like kinase-60 (SD-RLK) | plant immunity | 1 | 9 | 2 | 2 | 2 | 6 | 4 |
| OG0000569 | WRK70 | response to insect, fungus | 1 | 9 | 2 | 2 | 5 | 4 | 6 |
| OG0000627 | Probable serine/threonine-protein kinase PBL3 | plant defense | 1 | 6 | 3 | 4 | 3 | 3 | 4 |
| OG0000719 | EH domain-containing protein 1 | response to salt | 1 | 6 | 4 | 2 | 5 | 2 | 4 |
| OG0000728 | Zinc finger A20 and AN1 domain-containing stress-associated protein 4 | response to environmental stress | 1 | 2 | 4 | 3 | 3 | 4 | 3 |
| OG0000801 | BURP domain protein RD22 | response to salt stress | 1 | 12 | 2 | 3 | 2 | 2 | 3 |
| OG0000826 | Protein BRI1-5 ENHANCED 1 | response to absence of light | 1 | 2 | 3 | 4 | 5 | 4 | 4 |
| OG0000931 | Scarecrow-like protein | detoxification; response to xenobiotic stimulus | 1 | 5 | 5 | 3 | 3 | 2 | 3 |
| OG0000934 | R2R3-type MYB transcription factor | response to cold | 1 | 4 | 5 | 2 | 5 | 3 | 5 |
| OG0000963 | Potassium transporter 11 POT11 | plant growth; response to biotic and abiotic responses | 1 | 3 | 2 | 2 | 2 | 2 | 2 |
| OG0001088 | 3-hydroxyisobutyryl-CoA hydrolase 1, CHY1 | response to cold | 1 | 2 | 2 | 5 | 3 | 3 | 2 |
| OG0001180 | Protein SUPPRESSOR OF GENE SILENCING 3 | defense response to bacterium and virus | 1 | 3 | 2 | 4 | 3 | 2 | 5 |
| OG0001207 | NADPH-dependent alkenal/one oxidoreductase, chloroplastic | response to cold | 1 | 2 | 2 | 3 | 3 | 3 | 3 |
| OG0001222 | CUL3 | Regulation of cell death and immunity | 1 | 6 | 2 | 3 | 2 | 2 | 4 |
| OG0001440 | Carbohydrate-responsive element-binding protein (ChREBP) | response to high glucose | 1 | 3 | 3 | 2 | 3 | 3 | 3 |
| OG0001501 | Protein PIN-LIKES 3 PILS3 | response to auxin | 1 | 2 | 2 | 2 | 2 | 3 | 3 |
| OG0001609 | Probable WRKY transcription factor 75 | regulation of response to nutrient levels | 1 | 2 | 2 | 2 | 2 | 4 | 3 |
| OG0001658 | Peroxidase 45, PER5 | response to environmental stresses | 1 | 3 | 2 | 2 | 3 | 2 | 3 |
| OG0001663 | Potassium channel AKT1 | response to salt and drought stresses, | 1 | 2 | 3 | 2 | 3 | 2 | 3 |
| OG0001697 | Sugar transport protein 13 | response to salt, drought and ABA stresses | 1 | 3 | 2 | 3 | 4 | 3 | 2 |
| OG0001707 | Peroxisomal acyl-coenzyme A oxidase 1, ACX1 | response to fungus, wounding | 1 | 7 | 2 | 2 | 2 | 2 | 2 |
| OG0001768 | Uncharacterized protein At1g66480 | response to fungus | 1 | 2 | 2 | 2 | 2 | 2 | 6 |
| OG0001968 | CYP74A | response to fungus, wounding | 1 | 4 | 3 | 2 | 3 | 3 | 2 |
| OG0002044 | Transcription factor ICE1 | response to freezing;response to cold | 1 | 2 | 3 | 2 | 2 | 2 | 2 |
| OG0002128 | HSP20-like chaperone, acetyltransferase A | responses to salt stress and bacterial blight | 1 | 2 | 2 | 2 | 2 | 3 | 4 |
| OG0002150 | Inositol-pentakisphosphate 2-kinase, IPK1 | defense response to bacterium, fungi, verus | 1 | 2 | 2 | 2 | 2 | 2 | 2 |
| OG0002269 | Microtubule-destabilizing protein 60 | response to light stimulus | 1 | 3 | 2 | 2 | 2 | 2 | 2 |
| OG0002293 | Mitogen-activated protein kinase kinase kinase 3, MAPKKK3 | regulation of defense response to fungus | 1 | 3 | 2 | 2 | 2 | 2 | 4 |
| OG0002296 | Transcription factor TGA1 | defense response to bacterium | 1 | 2 | 2 | 2 | 2 | 2 | 2 |
| OG0002316 | GATA transcription factor 5 | regulation of some light-responsive genes | 1 | 3 | 2 | 2 | 2 | 2 | 2 |
| OG0002355 | Ribonuclease TUDOR 1, TSN1 | regulation during stress (e.g. salt and heat) | 1 | 2 | 2 | 4 | 2 | 2 | 2 |
| OG0002386 | BOI-related E3 ubiquitin-protein ligase 1, BRG1 | regulation of abiotic stress responses | 1 | 2 | 2 | 2 | 2 | 2 | 3 |
| OG0002462 | TIF3B | response to wounding | 1 | 2 | 2 | 2 | 2 | 2 | 3 |
| OG0002648 | SCP domain-containing protein | pathogenesis-related protein | 1 | 2 | 3 | 4 | 2 | 2 | 2 |
| OG0002658 | Triacylglycerol lipase 2 | defense response to insect | 1 | 2 | 2 | 2 | 3 | 2 | 3 |
| OG0002665 | TPP1 | improve abiotic stress tolerance | 1 | 4 | 2 | 2 | 2 | 2 | 2 |
| OG0002760 | PLAT domain-containing protein 2 | Involved in response to abiotic stress. | 1 | 3 | 2 | 2 | 2 | 2 | 2 |
| OG0002792 | Probable inactive poly [ADP-ribose] polymerase SRO2 | response to stress | 1 | 2 | 2 | 2 | 2 | 3 | 2 |
| OG0002794 | Two-component response regulator ARR6 | response to cytokinin | 1 | 2 | 2 | 2 | 3 | 2 | 2 |
| OG0002874 | Alkaline ceramidase, ACER | defenses responses against pathogenic bacteria | 1 | 3 | 2 | 2 | 2 | 2 | 2 |
| OG0002955 | MLO-like protein 1, MLO1 | pathogen defense | 1 | 2 | 2 | 2 | 2 | 2 | 4 |
| OG0002956 | Kinesin-like protein KIN-7D | regulation of defense response, immunity | 1 | 2 | 2 | 2 | 2 | 2 | 2 |
| OG0002992 | Patellin-6 | cellular response to auxin stimulus | 1 | 3 | 2 | 2 | 2 | 2 | 2 |
| OG0003125 | WRKY32 | immune response of plants | 1 | 2 | 2 | 2 | 2 | 2 | 3 |
| OG0003179 | NDR1/HIN1-like protein 6 | response to osmotic stress | 1 | 2 | 2 | 2 | 2 | 2 | 2 |
| OG0003270 | ARR11 | response to cytokinin | 1 | 2 | 3 | 2 | 2 | 2 | 2 |
| OG0003366 | (TIR-NBS-LRR class) | Disease resistance protein | 1 | 2 | 2 | 3 | 2 | 2 | 2 |
| OG0003382 | Copper-transporting ATPase RAN1 | response to ethylene | 1 | 2 | 2 | 2 | 2 | 2 | 3 |
| OG0003402 | Protein ABSCISIC ACID-INSENSITIVE 5 ABI5 | response to salt and drought | 1 | 2 | 2 | 2 | 2 | 2 | 2 |
| OG0003414 | E3 ubiquitin-protein ligase NLA | defense response to bacterium | 1 | 2 | 2 | 3 | 2 | 2 | 2 |
| OG0003429 | Calcium-transporting ATPase 4, plasma membrane-type, ACA4 | defense response to bacterium | 1 | 2 | 2 | 2 | 2 | 2 | 2 |
| OG0003448 | Phosphoenolpyruvate carboxylase kinase 2 | response to light stimulus | 1 | 2 | 2 | 2 | 2 | 2 | 2 |
| OG0003556 | Phosphatidate phosphatase PAH2 | response to phosphate starvation | 1 | 3 | 2 | 2 | 2 | 2 | 2 |
| OG0003956 | Calmodulin-binding receptor kinase CaMRLK | response to insect and osmotic stress | 1 | 2 | 2 | 2 | 2 | 2 | 2 |
| OG0003989 | Zinc finger protein BRUTUS， BTS | response to hypoxia | 1 | 2 | 2 | 2 | 2 | 2 | 2 |
| OG0004005 | Germin-like protein subfamily 3 member 4 | plant defense | 1 | 2 | 2 | 2 | 2 | 2 | 2 |
| OG0004071 | Phosphatidate phosphatase PAH1 | response to phosphate starvation | 1 | 2 | 2 | 2 | 2 | 2 | 2 |
| OG0004294 | Glucan endo-1,3-beta-glucosidase 5 | defense response | 1 | 2 | 2 | 2 | 2 | 2 | 2 |
| OG0004304 | Transcription factor bHLH63 | immune response | 1 | 2 | 2 | 2 | 2 | 2 | 2 |
| OG0000044 | LRR-repeat protein | plant immunity | 0 | 51 | 27 | 15 | 14 | 2 | 3 |
| OG0000072 | Receptor-like protein 6 | resistance against the fungal pathogens | 0 | 33 | 8 | 4 | 20 | 6 | 3 |
| OG0000234 | Bet_v_1 domain-containing protein | defense against pathogen infection | 0 | 6 | 8 | 8 | 17 | 3 | 10 |
| OG0000296 | LRR and NB-ARC domain disease resistance protein | disease resistance protein | 0 | 1 | 2 | 10 | 1 | 2 | 1 |
| OG0000303 | MLP-like protein 43 | defense against pathogen infection | 0 | 9 | 7 | 3 | 23 | 3 | 1 |
| OG0000553 | Kunitz type trypsin inhibitor | defense against insect pests | 0 | 6 | 4 | 1 | 12 | 4 | 6 |
| OG0000573 | Subtilisin-like protease SBT3.3 | plant immunity | 0 | 1 | 1 | 2 | 1 | 2 | 1 |
| OG0000613 | Organic cation/carnitine transporter 3 | response to cold | 0 | 6 | 6 | 1 | 3 | 4 | 2 |
| OG0000713 | Disease resistance protein (TIR-NBS-LRR class) | disease resistance against pathogen infection | 0 | 12 | 9 | 1 | 3 | 3 | 1 |
| OG0000731 | TIR-NBS-LRR type disease resistance protein | disease resistance against pathogen infection | 0 | 7 | 6 | 2 | 1 | 4 | 2 |
| OG0000896 | Haem peroxidase family protein | response to environmental stresses | 0 | 1 | 1 | 22 | 1 | 1 | 1 |
| OG0000927 | Zinc finger CCCH domain-containing protein 48 | response to biotic and abiotic stresses | 0 | 3 | 3 | 3 | 2 | 1 | 8 |
| OG0001217 | Kunitz type trypsin inhibitor | defense from insect pests and wounding | 0 | 3 | 7 | 2 | 1 | 7 | 4 |
| OG0001291 | Probable L-type lectin-domain containing receptor kinase VII.2 | defense response to bacterium and oomycetes | 0 | 5 | 4 | 3 | 4 | 2 | 1 |
| OG0001495 | NDR1/HIN1-like protein 10 | defense response to virus | 0 | 3 | 5 | 1 | 2 | 1 | 2 |
| OG0001805 | Cysteine protease XCP2 | defense response to bacterium | 0 | 6 | 2 | 3 | 1 | 3 | 5 |
| OG0002260 | Protein DETOXIFICATION | disease resistance | 0 | 2 | 2 | 4 | 1 | 3 | 3 |
| OG0002461 | C3HC4 type (RING finger) protein | stress response | 0 | 7 | 4 | 1 | 1 | 1 | 1 |
| OG0002578 | BON1-associated-like protein | disease resistance | 0 | 3 | 1 | 1 | 6 | 3 | 4 |
| OG0003031 | AAA-ATPase At2g18193 | response to stress | 0 | 4 | 3 | 3 | 3 | 1 | 2 |
| OG0004449 | 3-oxo-Delta(4,5)-steroid 5-beta-reductase VEP1 | response to wounding | 0 | 2 | 1 | 2 | 3 | 1 | 6 |
| OG0004450 | Xanthotoxin 5-hydroxylase CYP82C4 | response to iron ion | 0 | 3 | 1 | 5 | 1 | 2 | 1 |
| OG0004984 | Protein EIN4 | response to freezing | 0 | 2 | 3 | 1 | 2 | 2 | 2 |
| OG0006072 | Mitogen-activated protein kinase kinase kinase 5 | regulation of defense response to bacterium | 0 | 3 | 1 | 1 | 2 | 1 | 2 |
| OG0006407 | Zinc finger CCCH domain-containing protein 55 | response to abiotic stress | 0 | 1 | 1 | 1 | 1 | 2 | 1 |
| OG0007019 | Lectin receptor kinase | defense response to oomycetes, to bacterium | 0 | 1 | 1 | 6 | 2 | 1 | 1 |
| OG0007544 | Chalcone--flavanone isomerase 1, CHI1 | response to auxin, UVB | 0 | 2 | 3 | 1 | 2 | 2 | 2 |
| OG0008002 | HLH (helix-loop-helix) protein | defense to brown planthopper | 0 | 1 | 1 | 1 | 2 | 3 | 2 |
| OG0008646 | LEAF RUST 10 DISEASE-RESISTANCE LOCUS RECEPTOR-LIKE PROTEIN KINASE-like 1.2 (LRK12) | response to abiotic stress | 0 | 2 | 1 | 3 | 2 | 2 | 1 |
| OG0008936 | (E)-beta-ocimene synthase, chloroplastic | defense to herbivore | 0 | 2 | 1 | 1 | 4 | 1 | 2 |
| OG0009130 | Auxin responsive SAUR protein | response to auxin | 0 | 1 | 3 | 1 | 1 | 1 | 2 |
| OG0009227 | Zinc finger, RING/FYVE/PHD-type domain containing protein | abiotic stress tolerance | 0 | 2 | 1 | 1 | 1 | 1 | 3 |
| OG0009314 | Zinc finger, RING-type domain containing protein | resistance to both biotic and abiotic stress | 0 | 3 | 2 | 1 | 1 | 1 | 1 |
| OG0009689 | Glutathione S-transferase TCHQD | response to toxic substance | 0 | 1 | 1 | 1 | 2 | 1 | 2 |
| OG0009706 | Probable E3 ubiquitin-protein ligase XERICO | response to osmotic stress | 0 | 1 | 2 | 1 | 2 | 1 | 1 |
| OG0010372 | Protein EMSY-LIKE 3 | defense response to fungus | 0 | 2 | 1 | 1 | 1 | 1 | 1 |
| OG0010991 | Transcription factor bHLH120 | response to abiotic stresses | 0 | 1 | 2 | 2 | 2 | 2 | 1 |
| OG0011010 | IAA32 | response to auxin | 0 | 1 | 2 | 1 | 1 | 1 | 1 |
| OG0011119 | NB-ARC domain disease resistance protein | disease resistance | 0 | 1 | 1 | 1 | 1 | 1 | 1 |
| OG0011246 | Transcription factor HY5-like | response to UVB | 0 | 1 | 1 | 1 | 2 | 1 | 1 |
| OG0012198 | Phosphatidylinositol 4-kinase gamma 3 | response to salt, ABA | 0 | 1 | 1 | 1 | 1 | 1 | 1 |
| OG0012251 | Pectinesterase 1 PME1 | involved in salt stress response | 0 | 2 | 1 | 1 | 1 | 1 | 1 |
| OG0012439 | IAA4 | response to auxin | 0 | 1 | 2 | 1 | 2 | 1 | 2 |
| OG0012860 | Heat shock protein DnaJ | response to environmental stresses | 0 | 1 | 1 | 1 | 2 | 1 | 2 |
| OG0013118 | Putative transcription factor SBP family | resisting abiotic stress | 0 | 1 | 1 | 1 | 2 | 1 | 2 |
| OG0013368 | Transcription factor bHLH113 | response to abiotic stresses | 0 | 1 | 1 | 1 | 1 | 1 | 1 |
| OG0013563 | ERF020 | response to stress | 0 | 1 | 1 | 1 | 1 | 1 | 1 |
| OG0014215 | NIP | response to water deprivation | 0 | 1 | 2 | 1 | 1 | 1 | 1 |
| OG0014261 | Potassium transporter 1 POT1 | salt stress | 0 | 1 | 1 | 1 | 1 | 1 | 1 |
| OG0014376 | Leucine-rich receptor-like kinase family protein | plant immunity | 0 | 1 | 1 | 1 | 1 | 1 | 1 |
| OG0014507 | CPYC-type glutaredoxin | resistance to bacterial blight | 0 | 1 | 1 | 1 | 1 | 1 | 1 |
| OG0014517 | TOM1-like protein 2 | immune response | 0 | 1 | 1 | 1 | 1 | 1 | 1 |
| OG0014769 | ERF062 | response to stress | 0 | 1 | 1 | 1 | 1 | 1 | 1 |
| OG0015040 | MYB/HD-like transcription factor | response to gibberellin; response to salicylic acid | 0 | 1 | 1 | 1 | 1 | 1 | 1 |
| OG0015049 | F-box/LRR-like protein | plant immunity | 0 | 1 | 1 | 1 | 1 | 1 | 1 |
| OG0015364 | disease resistance response protein | disease resistance | 0 | 1 | 1 | 1 | 1 | 1 | 1 |
| OG0015377 | HVA22-like protein a | response to cold | 0 | 1 | 1 | 1 | 1 | 1 | 1 |
| OG0015527 | E3 ubiquitin-protein ligase AIRP2 | response to salt and drought stresses, ABA | 0 | 1 | 1 | 1 | 1 | 1 | 2 |
| OG0015740 | BAG family molecular chaperone regulator | stress responses | 0 | 1 | 1 | 1 | 1 | 1 | 1 |
| OG0016086 | bHLH | stress response | 0 | 1 | 1 | 1 | 1 | 1 | 1 |
| OG0016155 | Protein ASPARTIC PROTEASE IN GUARD CELL 1 ASPG1 | response to drought stress | 0 | 1 | 1 | 1 | 1 | 1 | 1 |
| OG0016278 | Transcription factor ILR3 | response to metal homeostasis changes. | 0 | 1 | 1 | 1 | 1 | 1 | 1 |
| OG0016306 | LRRNT_2 domain-containing protein | plant immunity | 0 | 1 | 1 | 1 | 1 | 1 | 1 |
| OG0016364 | Protein CANDIDATE G-PROTEIN COUPLED RECEPTOR 2 CAND2 | response to osmotic stress | 0 | 1 | 1 | 1 | 1 | 1 | 1 |

**Table S11.** Gene Ontology enrichment analysis of genes retained after the *Suriana*-specific WGD event.

| Category | GO ID | Go term description | Annotated | Significant | Expected | P-value |
| --- | --- | --- | --- | --- | --- | --- |
| **Biological process** | GO:0006468 | protein phosphorylation | 573 | 150 | 92.85 | 2.30E-10 |
|  | GO:0006793 | phosphorus metabolic process | 1726 | 351 | 279.68 | 3.10E-07 |
|  | GO:0006796 | phosphate-containing compound metabolic process | 1718 | 349 | 278.38 | 3.80E-07 |
|  | GO:0009719 | response to endogenous stimulus | 469 | 113 | 76 | 4.00E-06 |
|  | GO:0009725 | response to hormone | 465 | 112 | 75.35 | 4.50E-06 |
|  | GO:0071555 | cell wall organization | 212 | 55 | 34.35 | 0.00017 |
|  | GO:0050794 | regulation of cellular process | 1747 | 334 | 283.08 | 0.00019 |
|  | GO:0016049 | cell growth | 253 | 63 | 41 | 0.00021 |
|  | GO:0023052 | signaling | 730 | 153 | 118.29 | 0.00026 |
|  | GO:0007165 | signal transduction | 716 | 150 | 116.02 | 0.00031 |
|  | GO:0010119 | regulation of stomatal movement | 20 | 10 | 3.24 | 0.00047 |
|  | GO:0046856 | phosphatidylinositol dephosphorylation | 11 | 7 | 1.78 | 0.00052 |
|  | GO:0010289 | homogalacturonan biosynthetic process | 4 | 4 | 0.65 | 0.00069 |
|  | GO:0010394 | homogalacturonan metabolic process | 4 | 4 | 0.65 | 0.00069 |
|  | GO:0071396 | cellular response to lipid | 129 | 35 | 20.9 | 0.00106 |
|  | GO:0009742 | brassinosteroid mediated signaling pathway | 22 | 10 | 3.56 | 0.0012 |
|  | GO:0043401 | steroid hormone mediated signaling pathway | 22 | 10 | 3.56 | 0.0012 |
|  | GO:0048545 | response to steroid hormone | 22 | 10 | 3.56 | 0.0012 |
|  | GO:0071367 | cellular response to brassinosteroid stimulus | 22 | 10 | 3.56 | 0.0012 |
|  | GO:0071383 | cellular response to steroid hormone stimulus | 22 | 10 | 3.56 | 0.0012 |
|  | GO:0010118 | stomatal movement | 37 | 14 | 6 | 0.00123 |
|  | GO:0009755 | hormone-mediated signaling pathway | 234 | 56 | 37.92 | 0.00128 |
|  | GO:0010038 | response to metal ion | 230 | 55 | 37.27 | 0.00144 |
|  | GO:0065007 | biological regulation | 2269 | 414 | 367.66 | 0.00156 |
|  | GO:0032870 | cellular response to hormone stimulus | 236 | 56 | 38.24 | 0.00158 |
|  | GO:0071495 | cellular response to endogenous stimulus | 237 | 56 | 38.4 | 0.00175 |
|  | GO:0046686 | response to cadmium ion | 192 | 47 | 31.11 | 0.00186 |
|  | GO:1901700 | response to oxygen-containing compound | 629 | 129 | 101.92 | 0.00188 |
|  | GO:0009733 | response to auxin | 138 | 36 | 22.36 | 0.00192 |
|  | GO:0032957 | inositol trisphosphate metabolic process | 10 | 6 | 1.62 | 0.00208 |
|  | GO:0048829 | root cap development | 10 | 6 | 1.62 | 0.00208 |
|  | GO:0006813 | potassium ion transport | 43 | 15 | 6.97 | 0.00216 |
|  | GO:0009737 | response to abscisic acid | 165 | 41 | 26.74 | 0.00263 |
|  | GO:0045454 | cell redox homeostasis | 88 | 25 | 14.26 | 0.00266 |
|  | GO:0009116 | nucleoside metabolic process | 28 | 11 | 4.54 | 0.00287 |
|  | GO:0006801 | superoxide metabolic process | 14 | 7 | 2.27 | 0.00346 |
|  | GO:0007264 | small GTPase mediated signal transduction | 99 | 27 | 16.04 | 0.00347 |
|  | GO:0009932 | cell tip growth | 94 | 25 | 15.23 | 0.0068 |
|  | GO:0009967 | positive regulation of signal transduction | 9 | 5 | 1.46 | 0.00788 |
|  | GO:0023056 | positive regulation of signaling | 9 | 5 | 1.46 | 0.00788 |
|  | GO:0006811 | ion transport | 590 | 117 | 95.6 | 0.00914 |
|  | GO:0044070 | regulation of anion transport | 20 | 8 | 3.24 | 0.00943 |
|  | GO:0009738 | abscisic acid-activated signaling pathway | 55 | 16 | 8.91 | 0.01155 |
|  | GO:0010393 | galacturonan metabolic process | 46 | 14 | 7.45 | 0.01166 |
|  | GO:0045488 | pectin metabolic process | 46 | 14 | 7.45 | 0.01166 |
|  | GO:0050896 | response to stimulus | 2487 | 439 | 402.98 | 0.01292 |
|  | GO:0072657 | protein localization to membrane | 99 | 25 | 16.04 | 0.01342 |
|  | GO:0071215 | cellular response to abscisic acid stimulus | 56 | 16 | 9.07 | 0.0138 |
|  | GO:0051668 | localization within membrane | 101 | 25 | 16.37 | 0.01721 |
|  | GO:0042592 | homeostatic process | 194 | 43 | 31.44 | 0.01742 |
|  | GO:0048584 | positive regulation of response to stimulus | 22 | 8 | 3.56 | 0.01773 |
|  | GO:0043269 | regulation of ion transport | 67 | 18 | 10.86 | 0.01785 |
|  | GO:0019725 | cellular homeostasis | 163 | 37 | 26.41 | 0.01828 |
|  | GO:0051049 | regulation of transport | 72 | 19 | 11.67 | 0.01837 |
|  | GO:0006812 | cation transport | 419 | 84 | 67.89 | 0.01913 |
|  | GO:0035556 | intracellular signal transduction | 288 | 60 | 46.67 | 0.02102 |
|  | GO:0043478 | pigment accumulation in response to UV light | 32 | 10 | 5.19 | 0.02565 |
|  | GO:0030001 | metal ion transport | 237 | 50 | 38.4 | 0.02667 |
|  | GO:0006970 | response to osmotic stress | 266 | 55 | 43.1 | 0.03001 |
|  | GO:0009651 | response to salt stress | 239 | 50 | 38.73 | 0.03069 |
|  | GO:0005975 | carbohydrate metabolic process | 916 | 168 | 148.43 | 0.03757 |
|  | GO:0010200 | response to chitin | 63 | 16 | 10.21 | 0.04032 |
| **Molecular function** | GO:0004672 | protein kinase activity | 747 | 185 | 125.91 | 6.9E-09 |
|  | GO:0004674 | protein serine/threonine kinase activity | 504 | 133 | 84.95 | 2.1E-08 |
|  | GO:0016301 | kinase activity | 1038 | 237 | 174.96 | 1E-07 |
|  | GO:0004683 | calmodulin-dependent protein kinase activity | 66 | 28 | 11.12 | 8.4E-07 |
|  | GO:0022832 | voltage-gated channel activity | 37 | 19 | 6.24 | 1.5E-06 |
|  | GO:0005509 | calcium ion binding | 186 | 54 | 31.35 | 0.000022 |
|  | GO:0004721 | phosphoprotein phosphatase activity | 142 | 43 | 23.94 | 0.000049 |
|  | GO:0019904 | protein domain specific binding | 11 | 8 | 1.85 | 0.000065 |
|  | GO:0022836 | gated channel activity | 48 | 19 | 8.09 | 0.00015 |
|  | GO:0008308 | voltage-gated anion channel activity | 16 | 9 | 2.7 | 0.0004 |
|  | GO:0032555 | purine ribonucleotide binding | 1523 | 302 | 256.72 | 0.00054 |
|  | GO:0005253 | anion channel activity | 17 | 9 | 2.87 | 0.00071 |
|  | GO:0035639 | purine ribonucleoside triphosphate binding | 1510 | 298 | 254.52 | 0.00081 |
|  | GO:0032553 | ribonucleotide binding | 1550 | 305 | 261.27 | 0.00085 |
|  | GO:0022843 | voltage-gated cation channel activity | 21 | 10 | 3.54 | 0.00105 |
|  | GO:0000166 | nucleotide binding | 2232 | 424 | 376.22 | 0.00138 |
|  | GO:1901265 | nucleoside phosphate binding | 2232 | 424 | 376.22 | 0.00138 |
|  | GO:0016772 | transferase activity, transferring phosphorus-containing groups | 1301 | 258 | 219.3 | 0.00144 |
|  | GO:0005524 | ATP binding | 1314 | 258 | 221.49 | 0.00255 |
|  | GO:0097367 | carbohydrate derivative binding | 1583 | 305 | 266.83 | 0.00329 |
|  | GO:0015103 | inorganic anion transmembrane transporter activity | 57 | 18 | 9.61 | 0.00454 |
|  | GO:0015075 | ion transmembrane transporter activity | 461 | 99 | 77.71 | 0.00495 |
|  | GO:0004620 | phospholipase activity | 33 | 12 | 5.56 | 0.00555 |
|  | GO:0016298 | lipase activity | 59 | 18 | 9.94 | 0.00677 |
|  | GO:0043167 | ion binding | 3478 | 631 | 586.25 | 0.00697 |
|  | GO:0008320 | protein transmembrane transporter activity | 26 | 10 | 4.38 | 0.00706 |
|  | GO:0022884 | macromolecule transmembrane transporter activity | 26 | 10 | 4.38 | 0.00706 |
|  | GO:0140318 | protein transporter activity | 26 | 10 | 4.38 | 0.00706 |
|  | GO:0016757 | glycosyltransferase activity | 394 | 85 | 66.41 | 0.00779 |
|  | GO:0031406 | carboxylic acid binding | 27 | 10 | 4.55 | 0.00955 |
|  | GO:0004849 | uridine kinase activity | 10 | 5 | 1.69 | 0.01616 |
|  | GO:0030742 | GTP-dependent protein binding | 4 | 3 | 0.67 | 0.01671 |
|  | GO:0004774 | succinate-CoA ligase activity | 7 | 4 | 1.18 | 0.01831 |
|  | GO:0016841 | ammonia-lyase activity | 7 | 4 | 1.18 | 0.01831 |
|  | GO:0005249 | voltage-gated potassium channel activity | 18 | 7 | 3.03 | 0.02178 |
|  | GO:0035091 | phosphatidylinositol binding | 26 | 9 | 4.38 | 0.02186 |
|  | GO:0015035 | protein-disulfide reductase activity | 66 | 18 | 11.12 | 0.02233 |
|  | GO:0008081 | phosphoric diester hydrolase activity | 48 | 14 | 8.09 | 0.02361 |
|  | GO:0042393 | histone binding | 11 | 5 | 1.85 | 0.02558 |
|  | GO:0008289 | lipid binding | 106 | 26 | 17.87 | 0.02728 |
|  | GO:0005216 | ion channel activity | 116 | 28 | 19.55 | 0.0276 |
|  | GO:0015036 | disulfide oxidoreductase activity | 68 | 18 | 11.46 | 0.02982 |
|  | GO:0004602 | glutathione peroxidase activity | 8 | 4 | 1.35 | 0.0318 |
|  | GO:0008121 | ubiquinol-cytochrome-c reductase activit... | 8 | 4 | 1.35 | 0.0318 |
|  | GO:0035064 | methylated histone binding | 8 | 4 | 1.35 | 0.0318 |
|  | GO:0005267 | potassium channel activity | 20 | 7 | 3.37 | 0.03908 |
|  | GO:0004707 | MAP kinase activity | 16 | 6 | 2.7 | 0.03959 |
|  | GO:0015267 | channel activity | 121 | 28 | 20.4 | 0.0454 |
| **Cellular component** | GO:0005886 | plasma membrane | 1028 | 235 | 161.31 | 4E-11 |
|  | GO:0005773 | vacuole | 357 | 92 | 56.02 | 3.3E-07 |
|  | GO:0005618 | cell wall | 259 | 69 | 40.64 | 0.000003 |
|  | GO:0030312 | external encapsulating structure | 264 | 70 | 41.43 | 0.000003 |
|  | GO:0005794 | Golgi apparatus | 319 | 80 | 50.06 | 6.2E-06 |
|  | GO:0009505 | plant-type cell wall | 96 | 31 | 15.06 | 0.000036 |
|  | GO:0012505 | endomembrane system | 636 | 134 | 99.8 | 0.0001 |
|  | GO:0098588 | bounding membrane of organelle | 276 | 66 | 43.31 | 0.00019 |
|  | GO:0042719 | mitochondrial intermembrane space protein | 4 | 4 | 0.63 | 0.0006 |
|  | GO:0005730 | nucleolus | 196 | 48 | 30.76 | 0.00079 |
|  | GO:0012507 | ER to Golgi transport vesicle membrane | 10 | 6 | 1.57 | 0.00175 |
|  | GO:0030127 | COPII vesicle coat | 10 | 6 | 1.57 | 0.00175 |
|  | GO:0000502 | proteasome complex | 48 | 16 | 7.53 | 0.00188 |
|  | GO:0005774 | vacuolar membrane | 154 | 38 | 24.16 | 0.00231 |
|  | GO:0031974 | membrane-enclosed lumen | 346 | 74 | 54.29 | 0.00248 |
|  | GO:0043233 | organelle lumen | 346 | 74 | 54.29 | 0.00248 |
|  | GO:0070013 | intracellular organelle lumen | 346 | 74 | 54.29 | 0.00248 |
|  | GO:0031981 | nuclear lumen | 266 | 59 | 41.74 | 0.00282 |
|  | GO:1905369 | endopeptidase complex | 50 | 16 | 7.85 | 0.00302 |
|  | GO:0030134 | COPII-coated ER to Golgi transport vesicle | 11 | 6 | 1.73 | 0.00334 |
|  | GO:0005615 | extracellular space | 8 | 5 | 1.26 | 0.00349 |
|  | GO:0005741 | mitochondrial outer membrane | 23 | 9 | 3.61 | 0.00567 |
|  | GO:0005634 | nucleus | 1195 | 217 | 187.51 | 0.00636 |
|  | GO:0031968 | organelle outer membrane | 49 | 15 | 7.69 | 0.0064 |
|  | GO:1905368 | peptidase complex | 54 | 16 | 8.47 | 0.00699 |
|  | GO:0031410 | cytoplasmic vesicle | 162 | 37 | 25.42 | 0.01003 |
|  | GO:0097708 | intracellular vesicle | 162 | 37 | 25.42 | 0.01003 |
|  | GO:0005802 | trans-Golgi network | 86 | 22 | 13.49 | 0.01172 |
|  | GO:0019867 | outer membrane | 53 | 15 | 8.32 | 0.0138 |
|  | GO:0030658 | transport vesicle membrane | 14 | 6 | 2.2 | 0.01424 |
|  | GO:0098791 | Golgi apparatus subcompartment | 98 | 24 | 15.38 | 0.01505 |
|  | GO:0031982 | vesicle | 167 | 37 | 26.2 | 0.0162 |
|  | GO:0030135 | coated vesicle | 40 | 12 | 6.28 | 0.01656 |
|  | GO:0005758 | mitochondrial intermembrane space | 11 | 5 | 1.73 | 0.01906 |
|  | GO:0031970 | organelle envelope lumen | 11 | 5 | 1.73 | 0.01906 |
|  | GO:0005753 | mitochondrial proton-transporting ATP synthase complex | 23 | 8 | 3.61 | 0.0195 |
|  | GO:0033281 | TAT protein transport complex | 2 | 2 | 0.31 | 0.0246 |
|  | GO:0005838 | proteasome regulatory particle | 8 | 4 | 1.26 | 0.02487 |
|  | GO:0009897 | external side of plasma membrane | 8 | 4 | 1.26 | 0.02487 |
|  | GO:0022624 | proteasome accessory complex | 8 | 4 | 1.26 | 0.02487 |
|  | GO:0009524 | phragmoplast | 24 | 8 | 3.77 | 0.02533 |
|  | GO:0030120 | vesicle coat | 33 | 10 | 5.18 | 0.02574 |
|  | GO:0005819 | spindle | 29 | 9 | 4.55 | 0.02892 |
|  | GO:0030662 | coated vesicle membrane | 34 | 10 | 5.34 | 0.03151 |
|  | GO:0005654 | nucleoplasm | 64 | 16 | 10.04 | 0.03535 |
|  | GO:0005576 | extracellular region | 322 | 62 | 50.53 | 0.04535 |

**Table S12.** Number of transcription factors detected in *S. maritima* and the number of retained duplicates after lineage-specific WGD. The transcription factor in bold indicate they have experienced expansion.

| Transcription factor | No. of genes | No. of WGD retained transcriptor factors | Proportion |
| --- | --- | --- | --- |
| AP2 | 17 | 7 | 41.2% |
| **ARF** | 20 | 4 | 20.0% |
| **ARR-B** | 10 | 6 | 60.0% |
| **B3** | 33 | 4 | 12.1% |
| **BBR-BPC** | 5 | 2 | 40.0% |
| **BES1** | 4 | 0 | 0 |
| **bHLH** | 119 | 23 | 19.3% |
| **bZIP** | 67 | 26 | 38.8% |
| **C2H2** | 114 | 36 | 31.6% |
| **C3H** | 51 | 12 | 23.5% |
| CAMTA | 5 | 2 | 40.0% |
| **CO-like** | 11 | 0 | 0 |
| **CPP** | 7 | 2 | 28.6% |
| DBB | 2 | 1 | 50.0% |
| **Dof** | 26 | 6 | 23.1% |
| **E2F/DP** | 8 | 0 | 0 |
| **EIL** | 6 | 0 | 0 |
| **ERF** | 115 | 27 | 23.5% |
| **FAR1** | 19 | 0 | 0 |
| **G2-like** | 40 | 6 | 15.0% |
| **GATA** | 31 | 14 | 45.2% |
| GeBP | 5 | 0 | 0 |
| GRAS | 42 | 8 | 19.0% |
| **GRF** | 10 | 4 | 40.0% |
| HB-other | 8 | 0 | 0 |
| HB-PHD | 8 | 0 | 0 |
| **HD-ZIP** | 40 | 14 | 35.0% |
| HRT-like | 1 | 0 | 0 |
| **HSF** | 19 | 4 | 21.1% |
| **LBD** | 39 | 8 | 20.5% |
| LFY | 1 | 0 | 0 |
| LSD | 4 | 0 | 0 |
| **MIKC_MADS** | 38 | 16 | 42.1% |
| **M-type_MADS** | 20 | 3 | 15.0% |
| **MYB** | 135 | 43 | 31.9% |
| **MYB_related** | 49 | 6 | 12.2% |
| **NAC** | 92 | 24 | 26.1% |
| NF-X1 | 2 | 0 | 0 |
| NF-YA | 8 | 2 | 25.0% |
| **NF-YB** | 22 | 4 | 18.2% |
| **NF-YC** | 12 | 4 | 33.3% |
| Nin-like | 12 | 0 | 0 |
| **RAV** | 2 | 0 | 0 |
| S1Fa-like | 2 | 0 | 0 |
| SAP | 1 | 0 | 0 |
| **SBP** | 17 | 2 | 11.8% |
| SRS | 6 | 0 | 0 |
| STAT | 1 | 0 | 0 |
| TALE | 17 | 0 | 0 |
| **TCP** | 21 | 8 | 38.1% |
| **Trihelix** | 27 | 6 | 22.2% |
| VOZ | 2 | 0 | 0 |
| Whirly | 2 | 0 | 0 |
| **WOX** | 12 | 2 | 16.7% |
| **WRKY** | 69 | 20 | 29.0% |
| YABBY | 7 | 2 | 28.6% |
| **ZF-HD** | 15 | 6 | 40.0% |
| **OFP** | 20 | 2 | 10.0% |
| **PLATZ** | 11 | 4 | 36.4% |

**Table S13.** Number of retained genes after lineage-specific WGD in stress-related gene families.

| Gene family | No. of genes | No. of WGD retained genes | Proportion |
| --- | --- | --- | --- |
| AHK5 | 8 | 2 | 25.0% |
| ALMT12 | 11 | 2 | 18.2% |
| APX1 | 7 | 2 | 28.6% |
| ANN1 | 10 | 4 | 40.0% |
| AKT1 | 8 | 4 | 50.0% |
| CAMTA1 | 6 | 2 | 33.3% |
| DHN1 | 8 | 2 | 25.0% |
| GPX | 7 | 4 | 57.1% |
| MSD | 3 | 2 | 66.7% |
| MDAR | 5 | 2 | 40.0% |
| NHX | 7 | 4 | 57.1% |
| CIPK | 23 | 10 | 43.5% |
| CML | 25 | 7 | 28.0% |
| CDPK | 32 | 18 | 56.3% |
| CBL | 10 | 6 | 60.0% |
| MAPK | 15 | 6 | 40.0% |
| MKK | 7 | 3 | 42.9% |
| HAK5 | 21 | 8 | 38.1% |
| NPF | 51 | 10 | 19.6% |
| NIP | 9 | 2 | 22.2% |
| TIP | 11 | 4 | 36.4% |
| PIP | 13 | 4 | 30.8% |
| SAP | 9 | 5 | 55.6% |
| SLAC | 6 | 2 | 33.3% |
| PKS | 19 | 8 | 42.1% |
| H+ATPase | 13 | 6 | 46.2% |
| 14-3-3 | 11 | 8 | 72.7% |

**Table S14.** Details of single-copy orthologous genes under positive selection in the genome of *Suriana maritima*. We listed the functions for those genes probably involved in responses to drought and salt stresses.

| Gene | Alternative hypothesis | Null hypothesis | *P*-value | Gene description | Gene name | Function |
| --- | --- | --- | --- | --- | --- | --- |
|  | lnL | lnL |  |  |  |  |
| EVM0013233 | -2357.990954 | -2366.181696 | 5.1788E-05 | Putative L-ascorbate peroxidase 6 APX6 | APX6 | antioxidation |
| EVM0014864 | -6694.027765 | -6704.191464 | 6.526E-06 | L-ascorbate oxidase | ASO | antioxidation |
| EVM0014876 | -6598.802196 | -6601.146743 | 0.0304 | 30-kDa cleavage and polyadenylation specificity factor 30 | CPSF30 | antioxidation |
| EVM0016764 | -2074.203019 | -2076.968842 | 0.0187 | Glutaredoxin-C5, chloroplastic | GRXC5 | antioxidation |
| EVM0013464 | -2390.829474 | -2394.965547 | 0.0040 | 1-Cys peroxiredoxin | PER1 | antioxidation |
| EVM0010895 | -3483.277552 | -3486.158833 | 0.0164 | Peroxidase 11 | PER11 | antioxidation |
| EVM0007011 | -2868.021984 | -2870.648947 | 0.0219 | Peroxidase 40 | PER40 | antioxidation |
| EVM0020589 | -3672.282636 | -3675.296562 | 0.0141 | Peroxidase 9 | PER9 | antioxidation |
| EVM0004462 | -2146.112242 | -2148.268153 | 0.0378 | Sirohydrochlorin ferrochelatase, chloroplastic | SirB | antioxidation |
| EVM0004689 | -4886.522599 | -4889.846863 | 0.0099 | NF-X1-type zinc finger protein NFXL2 | NFXL2 | antioxidation, response to salt and oxidative stresses |
| EVM0011559 | -4924.95562 | -4932.332464 | 0.0001 | NADPH:adrenodoxin oxidoreductase, mitochondrial | MFDR | antioxidation |
| EVM0021888 | -3391.003671 | -3394.866385 | 0.0054 | Copper chaperone for superoxide dismutase, chloroplastic/cytosolic | CCS | antioxidation |
| EVM0009292 | -3242.999452 | -3247.07929 | 0.0043 | NADH dehydrogenase (ubiquinone) complex I, assembly factor 6 | Ndufaf6 | antioxidation |
| EVM0005693 | -8766.947696 | -8768.924854 | 0.0468 | Respiratory burst oxidase homolog protein F | RBOHF | antioxidation |
| EVM0008077 | -10700.78906 | -10698.72675 | 0.0423 | Receptor protein-tyrosine kinase CEPR1 | CEPR1 | antioxidation, flavonoid biosynthesis |
| EVM0005526 | -4104.289471 | -4108.470136 | 0.0038 | Flavonol synthase/flavanone 3-hydroxylase | F3H | antioxidation, flavonoid biosynthesis |
| EVM0009889 | -4985.237641 | -4990.51167 | 0.0012 | Probable sodium/metabolite cotransporter BASS3, chloroplastic | BASS3 | sodium ion transport |
| EVM0003461 | -9840.995063 | -9845.524529 | 0.0026 | Glutamate receptor 3.7 | Gria3.7 | ion channel |
| EVM0005964 | -4506.928448 | -4509.143763 | 0.0353 | Two-pore potassium channel 5 | KCO5 | ion channel |
| EVM0002372 | -8876.117167 | -8878.429969 | 0.0315 | Protein CATION/H+ EXCHANGER 15 | CHX15 | ion transport |
| EVM0010615 | -4306.222212 | -4309.115721 | 0.0161 | Magnesium Transporter 10 | MGT10 | ion transport |
| EVM0021916 | -4299.038411 | -4302.605267 | 0.0076 | Magnesium Transporter 6 | MGT6 | ion transport |
| EVM0005762 | -5328.640417 | -5332.426459 | 0.0059 | K(+) efflux antiporter 5 | KEA5 | osmotic adjustment |
| EVM0021499 | -4930.657051 | -4932.795231 | 0.0386 | Aluminum-activated citrate transporter | MATE42 | transport |
| EVM0000257 | -3302.642542 | -3309.881085 | 0.0001 | Metal tolerance protein C1 | MTPC1 | transport |
| EVM0017685 | -6304.839972 | -6307.16635 | 0.0310 | Protein NRT1/ PTR FAMILY 5.1 | NPF5.1 | transport |
| EVM0004821 | -7732.555519 | -7734.542669 | 0.0462 | Oligopeptide transporter 7 | OPT7 | transport |
| EVM0016774 | -5002.01107 | -5007.68981 | 0.0008 | Probable polyol transporter 4 | PLT4 | transport |
| EVM0005409 | -6905.087834 | -6907.64227 | 0.0238 | 26S rRNA (cytosine-C(5))-methyltransferase NOP2B | TRM4d | transport |
| EVM0001984 | -6631.235616 | -6633.592854 | 0.0299 | TSET complex member tstC | tstC | transport |
| EVM0010580 | -36470.60527 | -36458.90493 | 1.315E-06 | Intermembrane lipid transfer protein vps13A | vps13A | transport |
| EVM0013720 | -3670.872072 | -3668.554551 | 0.0313 | Xylulose 5-phosphate/phosphate translocator, chloroplastic | XPT | transport |
| EVM0016391 | -7739.642225 | -7747.934366 | 4.6535E-05 | Metal-nicotianamine transporter YSL1 | YSL1 | transport |
| EVM0006402 | -4532.670298 | -4536.181494 | 0.0080 | Protein ELC | ELC | transport |
| EVM0005670 | -4388.385539 | -4394.11662 | 0.0007 | Tetracycline resistance protein, class A | TetA | transport |
| EVM0004501 | -24021.09428 | -24041.49889 | 0 | Protein TRANSLOCATED PROMOTER REGION | TPR | transport |
| EVM0020965 | -2873.139897 | -2876.000684 | 0.0168 | Non-specific lipid transfer protein GPI-anchored 2 | LTPG2 | transport |
| EVM0020788 | -1682.320306 | -1684.305403 | 0.0463 | Chloroplastic outer envelope pore protein of 16 kDa 4 | OEP16-4 | transport |
| EVM0011300 | -7477.9383 | -7479.930706 | 0.0459 | MICOS complex subunit MIC60, mitochondrial | MIC60 | transport |
| EVM0009981 | -7383.382924 | -7391.030928 | 9.1911E-05 | Probable S-adenosylmethionine carrier 2, chloroplastic | SAMC2 | transport |
| EVM0016375 | -2479.162056 | -2483.999502 | 0.0019 | Protein ARV 1 | ARV1 | transport |
| EVM0006854 | -3593.375349 | -3601.317457 | 6.7339E-05 | Late embryogenesis abundant protein D-29 | LEA D-29 | protectant protein |
| EVM0001942 | -3329.05146 | -3332.969862 | 0.0051 | AP2-like ethylene-responsive transcription factor AIL1 | AIL1 | transcription factor |
| EVM0010893 | -1203.91026 | -1207.084872 | 0.0117 | Transcription factor bHLH110 | bHLH110 | transcription factor |
| EVM0020355 | -1278.739379 | -1281.391316 | 0.0213 | transcription factor bHLH151 | bHLH151 | transcription factor |
| EVM0006371 | -7560.179712 | -7562.618711 | 0.0272 | bHLH transcription factor | bHLH156 | transcription factor |
| EVM0015667 | -3140.145097 | -3143.432765 | 0.0103 | ABSCISIC ACID-INSENSITIVE 5-like protein 2 | bZIP66 | transcription factor |
| EVM0008195 | -2680.876232 | -2684.225156 | 0.0097 | bZIP transcription factor 66 | bZIP66 | transcription factor |
| EVM0004247 | -4481.464942 | -4484.697969 | 0.0110 | E2F transcription factor-2 | E2F2 | transcription factor |
| EVM0007385 | -7054.982133 | -7051.443554 | 0.0078 | Homeobox-leucine zipper protein ROC3 | HD-ZIP ROC3 | transcription factor |
| EVM0009935 | -3882.405689 | -3885.411329 | 0.0142 | Heat shock transcription factor 3 | Hstf3 | transcription factor |
| EVM0008043 | -1545.477266 | -1547.499898 | 0.0443 | Transcription factor HY5 | HY5 | transcription factor |
| EVM0004763 | -2862.26938 | -2871.824668 | 1.2336E-05 | Transcription factor MYB52 | MYB52 | transcription factor |
| EVM0021320 | -3692.35029 | -3697.050993 | 0.0022 | Transcription factor TCP19 | TCP19 | transcription factor |
| EVM0015457 | -2708.638734 | -2710.734983 | 0.0406 | Trihelix transcription factor GT-3b | Trihelix | transcription factor |
| EVM0000085 | -6688.494836 | -6691.877379 | 0.0093 | Probable WRKY transcription factor 2 | WRKY2 | transcription factor |
| EVM0000493 | -4972.415635 | -4978.879387 | 0.0003 | Probable WRKY transcription factor 9 | WRKY9 | transcription factor |
| EVM0012642 | -2987.069614 | -2990.610785 | 0.0078 | E3 ubiquitin-protein ligase SDIR1 | SDIR1 | abscisic acid-related stress signal transduction |
| EVM0018351 | -8445.122983 | -8461.111653 | 1.6E-08 | E3 ubiquitin-protein ligase SDIR1 | SDIR1 | abscisic acid-related stress signal transduction |
| EVM0018362 | -2061.716256 | -2063.651937 | 0.0491 | E3 ubiquitin-protein ligase RHA2A | RHA2A | abscisic acid-related stress signal transduction |
| EVM0015085 | -2659.018783 | -2662.561308 | 0.0078 | Serine/threonine-protein phosphatase PP1 isozyme 4 | TOPP1 | signal transduction |
| EVM0018918 | -3673.45168 | -3681.419132 | 6.5559E-05 | Probable calcium-binding protein CML10 | CML10 | signal transduction |
| EVM0010298 | -5512.089755 | -5517.403234 | 0.0011 | Xylan glycosyltransferase MUCI21 | MUCI21 | cell wall organization |
| EVM0015904 | -4077.998765 | -4081.22519 | 0.0111 | COBRA-like protein 4 | COBL4 | cell wall organization |
| EVM0008759 | -3200.798204 | -3203.846999 | 0.0135 | Endoglucanase 9 | GLU1 | cell wall assembly |
| EVM0017023 | -2760.451807 | -2762.844251 | 0.0287 | Glycine-rich RNA-binding protein RZ1B | RZ1B | response to water deprivation |
| EVM0016760 | -6738.263816 | -6741.036363 | 0.0185 | Tyrosyl-DNA phosphodiesterase 1 | TDP1 | DNA repair |
| EVM0015506 | -13862.37827 | -13867.50051 | 0.0014 | DNA repair protein UVH3 | UVH3 | DNA repair |
| EVM0010901 | -5726.357192 | -5729.755669 | 0.0091 | Crossover junction endonuclease EME1B | EME1B | DNA repair |
| EVM0005101 | -4897.742455 | -4902.474296 | 0.0021 | WD repeat-containing protein ATCSA-1 | ATCSA-1 | DNA repair |

**Table S15.** Gene Ontology enrichment analysis of positively selected genes of single-copy orthologous genes.

| Category | GO ID | Go term description | Annotated | Significant | Expected | P-value |
| --- | --- | --- | --- | --- | --- | --- |
| **Biological process** | GO:0009668 | plastid membrane organization | 105 | 11 | 2.81 | 0.00011 |
|  | GO:0010027 | thylakoid membrane organization | 105 | 11 | 2.81 | 0.00011 |
|  | GO:0006396 | RNA processing | 443 | 26 | 11.85 | 0.00013 |
|  | GO:0061024 | membrane organization | 121 | 11 | 3.24 | 0.00038 |
|  | GO:0009657 | plastid organization | 197 | 14 | 5.27 | 0.00082 |
|  | GO:0031537 | regulation of anthocyanin metabolic process | 9 | 3 | 0.24 | 0.00141 |
|  | GO:0042440 | pigment metabolic process | 211 | 14 | 5.64 | 0.00158 |
|  | GO:0031050 | dsRNA processing | 48 | 6 | 1.28 | 0.00165 |
|  | GO:0090304 | nucleic acid metabolic process | 1664 | 63 | 44.51 | 0.00194 |
|  | GO:1903311 | regulation of mRNA metabolic process | 3 | 2 | 0.08 | 0.0021 |
|  | GO:0060255 | regulation of macromolecule metabolic process | 892 | 38 | 23.86 | 0.00259 |
|  | GO:0019252 | starch biosynthetic process | 91 | 8 | 2.43 | 0.00294 |
|  | GO:0010629 | negative regulation of gene expression | 156 | 11 | 4.17 | 0.00309 |
|  | GO:0034660 | ncRNA metabolic process | 252 | 15 | 6.74 | 0.00314 |
|  | GO:0000023 | maltose metabolic process | 74 | 7 | 1.98 | 0.00353 |
|  | GO:0043412 | macromolecule modification | 1448 | 55 | 38.73 | 0.00374 |
|  | GO:0048367 | shoot system development | 483 | 23 | 12.92 | 0.00517 |
|  | GO:0005982 | starch metabolic process | 106 | 8 | 2.84 | 0.00742 |
|  | GO:0005984 | disaccharide metabolic process | 106 | 8 | 2.84 | 0.00742 |
|  | GO:0043170 | macromolecule metabolic process | 4089 | 129 | 109.37 | 0.0076 |
|  | GO:0009962 | regulation of flavonoid biosynthetic process | 16 | 3 | 0.43 | 0.00818 |
|  | GO:0046483 | heterocycle metabolic process | 2325 | 79 | 62.19 | 0.00927 |
|  | GO:0009902 | chloroplast relocation | 49 | 5 | 1.31 | 0.00962 |
|  | GO:0051667 | establishment of plastid localization | 49 | 5 | 1.31 | 0.00962 |
|  | GO:0006979 | response to oxidative stress | 207 | 12 | 5.54 | 0.00966 |
|  | GO:0010374 | stomatal complex development | 70 | 6 | 1.87 | 0.01085 |
|  | GO:0010207 | photosystem II assembly | 92 | 7 | 2.46 | 0.01148 |
|  | GO:0010351 | lithium ion transport | 19 | 3 | 0.51 | 0.01335 |
|  | GO:0009908 | flower development | 307 | 15 | 8.21 | 0.0179 |
|  | GO:0015996 | chlorophyll catabolic process | 38 | 4 | 1.02 | 0.01809 |
|  | GO:0046149 | pigment catabolic process | 38 | 4 | 1.02 | 0.01809 |
|  | GO:0033015 | tetrapyrrole catabolic process | 40 | 4 | 1.07 | 0.02149 |
|  | GO:0009235 | cobalamin metabolic process | 1 | 1 | 0.03 | 0.02675 |
|  | GO:0009236 | cobalamin biosynthetic process | 1 | 1 | 0.03 | 0.02675 |
|  | GO:0019418 | sulfide oxidation | 1 | 1 | 0.03 | 0.02675 |
|  | GO:0031440 | regulation of mRNA 3'-end processing | 1 | 1 | 0.03 | 0.02675 |
|  | GO:0034756 | regulation of iron ion transport | 1 | 1 | 0.03 | 0.02675 |
|  | GO:0051792 | medium-chain fatty acid biosynthetic process | 1 | 1 | 0.03 | 0.02675 |
|  | GO:0060776 | simple leaf morphogenesis | 1 | 1 | 0.03 | 0.02675 |
|  | GO:0061013 | regulation of mRNA catabolic process | 1 | 1 | 0.03 | 0.02675 |
|  | GO:0070453 | regulation of heme biosynthetic process | 1 | 1 | 0.03 | 0.02675 |
|  | GO:0005983 | starch catabolic process | 10 | 2 | 0.27 | 0.02784 |
|  | GO:0006644 | phospholipid metabolic process | 271 | 13 | 7.25 | 0.03015 |
|  | GO:0061458 | reproductive system development | 604 | 24 | 16.16 | 0.03325 |
|  | GO:0009963 | positive regulation of flavonoid biosynthesis | 11 | 2 | 0.29 | 0.03343 |
|  | GO:0046148 | pigment biosynthetic process | 166 | 9 | 4.44 | 0.03412 |
|  | GO:0006891 | intra-Golgi vesicle-mediated transport | 27 | 3 | 0.72 | 0.03447 |
|  | GO:0009251 | glucan catabolic process | 12 | 2 | 0.32 | 0.03942 |
|  | GO:0044247 | cellular polysaccharide catabolic process | 12 | 2 | 0.32 | 0.03942 |
|  | GO:0009888 | tissue development | 401 | 17 | 10.73 | 0.04075 |
|  | GO:0048449 | floral organ formation | 49 | 4 | 1.31 | 0.04142 |
|  | GO:0009909 | regulation of flower development | 121 | 7 | 3.24 | 0.04347 |
|  | GO:0003006 | developmental process involved in reproduction | 718 | 27 | 19.2 | 0.04476 |
|  | GO:0009812 | flavonoid metabolic process | 73 | 5 | 1.95 | 0.04544 |
|  | GO:0016109 | tetraterpenoid biosynthetic process | 73 | 5 | 1.95 | 0.04544 |
|  | GO:0016117 | carotenoid biosynthetic process | 73 | 5 | 1.95 | 0.04544 |
| **Molecular function** | GO:0016866 | intramolecular transferase activity | 46 | 6 | 1.13 | 0.00085 |
|  | GO:0009982 | pseudouridine synthase activity | 11 | 3 | 0.27 | 0.00209 |
|  | GO:0016868 | intramolecular transferase activity, phosphotransferases | 14 | 3 | 0.34 | 0.00436 |
|  | GO:0008374 | O-acyltransferase activity | 29 | 4 | 0.71 | 0.00521 |
|  | GO:0030247 | polysaccharide binding | 16 | 3 | 0.39 | 0.00648 |
|  | GO:0008135 | translation factor activity, RNA binding | 113 | 8 | 2.78 | 0.00662 |
|  | GO:0003676 | nucleic acid binding | 1568 | 53 | 38.52 | 0.00836 |
|  | GO:0004366 | glycerol-3-phosphate O-acyltransferase activity | 7 | 2 | 0.17 | 0.01164 |
|  | GO:0016149 | translation release factor activity, codon specific | 7 | 2 | 0.17 | 0.01164 |
|  | GO:0016853 | isomerase activity | 210 | 11 | 5.16 | 0.01458 |
|  | GO:0003747 | translation release factor activity | 10 | 2 | 0.25 | 0.02376 |
|  | GO:0008079 | translation termination factor activity | 10 | 2 | 0.25 | 0.02376 |
|  | GO:0031072 | heat shock protein binding | 45 | 4 | 1.11 | 0.02411 |
|  | GO:0000987 | cis-regulatory region sequence-specific DNA binding | 1 | 1 | 0.02 | 0.02457 |
|  | GO:0003730 | mRNA 3'-UTR binding | 1 | 1 | 0.02 | 0.02457 |
|  | GO:0003846 | 2-acylglycerol O-acyltransferase activit... | 1 | 1 | 0.02 | 0.02457 |
|  | GO:0004147 | dihydrolipoamide branched chain acyltran... | 1 | 1 | 0.02 | 0.02457 |
|  | GO:0004344 | glucose dehydrogenase activity | 1 | 1 | 0.02 | 0.02457 |
|  | GO:0004622 | lysophospholipase activity | 1 | 1 | 0.02 | 0.02457 |
|  | GO:0004679 | AMP-activated protein kinase activity | 1 | 1 | 0.02 | 0.02457 |
|  | GO:0004846 | urate oxidase activity | 1 | 1 | 0.02 | 0.02457 |
|  | GO:0008672 | 2-dehydro-3-deoxyglucarate aldolase activity | 1 | 1 | 0.02 | 0.02457 |
|  | GO:0008679 | 2-hydroxy-3-oxopropionate reductase activity | 1 | 1 | 0.02 | 0.02457 |
|  | GO:0008691 | 3-hydroxybutyryl-CoA dehydrogenase activity | 1 | 1 | 0.02 | 0.02457 |
|  | GO:0008837 | diaminopimelate epimerase activity | 1 | 1 | 0.02 | 0.02457 |
|  | GO:0009884 | cytokinin receptor activity | 1 | 1 | 0.02 | 0.02457 |
|  | GO:0016663 | oxidoreductase activity, acting on other nitrogenous compounds as donors, oxygen as acceptor | 1 | 1 | 0.02 | 0.02457 |
|  | GO:0016673 | oxidoreductase activity, acting on a sulfur group of donors, iron-sulfur protein as acceptor | 1 | 1 | 0.02 | 0.02457 |
|  | GO:0018489 | vanillate monooxygenase activity | 1 | 1 | 0.02 | 0.02457 |
|  | GO:0035184 | histone threonine kinase activity | 1 | 1 | 0.02 | 0.02457 |
|  | GO:0047936 | glucose 1-dehydrogenase [NAD(P)] activity | 1 | 1 | 0.02 | 0.02457 |
|  | GO:0050311 | sulfite reductase (ferredoxin) activity | 1 | 1 | 0.02 | 0.02457 |
|  | GO:0003844 | 1,4-alpha-glucan branching enzyme activity | 2 | 1 | 0.05 | 0.04854 |
|  | GO:0003935 | GTP cyclohydrolase II activity | 2 | 1 | 0.05 | 0.04854 |
|  | GO:0004615 | phosphomannomutase activity | 2 | 1 | 0.05 | 0.04854 |
|  | GO:0004789 | thiamine-phosphate diphosphorylase activity | 2 | 1 | 0.05 | 0.04854 |
|  | GO:0005034 | osmosensor activity | 2 | 1 | 0.05 | 0.04854 |
|  | GO:0008686 | 3,4-dihydroxy-2-butanone-4-phosphate synthase activity | 2 | 1 | 0.05 | 0.04854 |
|  | GO:0015271 | outward rectifier potassium channel activity | 2 | 1 | 0.05 | 0.04854 |
|  | GO:0016532 | superoxide dismutase copper chaperone activity | 2 | 1 | 0.05 | 0.04854 |
|  | GO:0030267 | glyoxylate reductase (NADP+) activity | 2 | 1 | 0.05 | 0.04854 |
|  | GO:0034256 | chlorophyll(ide) b reductase activity | 2 | 1 | 0.05 | 0.04854 |
| **Cellular component** | GO:0009532 | plastid stroma | 439 | 23 | 10.98 | 0.00056 |
|  | GO:0009570 | chloroplast stroma | 420 | 22 | 10.51 | 0.00075 |
|  | GO:0009941 | chloroplast envelope | 354 | 17 | 8.86 | 0.00721 |
|  | GO:0009654 | photosystem II oxygen evolving complex | 19 | 3 | 0.48 | 0.01111 |
|  | GO:0009507 | chloroplast | 1739 | 57 | 43.51 | 0.01258 |
|  | GO:0098807 | chloroplast thylakoid membrane protein complex | 10 | 2 | 0.25 | 0.02454 |
|  | GO:0009341 | beta-galactosidase complex | 1 | 1 | 0.03 | 0.02502 |
|  | GO:0043036 | starch grain | 1 | 1 | 0.03 | 0.02502 |
|  | GO:0043596 | nuclear replication fork | 1 | 1 | 0.03 | 0.02502 |
|  | GO:0043601 | nuclear replisome | 1 | 1 | 0.03 | 0.02502 |
|  | GO:0009543 | chloroplast thylakoid lumen | 47 | 4 | 1.18 | 0.0293 |
|  | GO:0019898 | extrinsic component of membrane | 47 | 4 | 1.18 | 0.0293 |
|  | GO:0031978 | plastid thylakoid lumen | 47 | 4 | 1.18 | 0.0293 |
|  | GO:0009536 | plastid | 1868 | 58 | 46.73 | 0.03393 |
|  | GO:0000151 | ubiquitin ligase complex | 100 | 6 | 2.5 | 0.03889 |

**Table S16.** Detailed annotation of up-regulated genes under drought treatment.

| *Suriana maritima* | *Arabidopsis thaliana* | Gene name | Gene description | Function |
| --- | --- | --- | --- | --- |
| EVM0004656 | AT3G09640 | APX2 | L-ascorbate peroxidase 2, cytosolic | antioxidation |
| EVM0002107 | AT5G21482 | CKX7 | Cytokinin dehydrogenase 7 | antioxidation |
| EVM0018754 | AT4G21490 | NDB3 | External alternative NAD(P)H-ubiquinone oxidoreductase B3, mitochondrial | antioxidation |
| EVM0000110 | AT5G17230 | PSY | Phytoene synthase, chloroplastic | antioxidation |
| EVM0017496 | AT3G57520 | RFS2 | Probable galactinol--sucrose galactosyltransferase 2 | antioxidation |
| EVM0009351 | AT1G78380 | GSTU19 | Glutathione S-transferase U19 | antioxidation |
| EVM0004656 | AT1G07890 | APX1 | L-ascorbate peroxidase 1, cytosolic | antioxidation |
| EVM0009819 | AT4G04330 | RBCX1 | Chaperonin-like RBCX protein 1, chloroplastic | chaperones, |
| EVM0009968 | AT1G74310 | ClpB1 | Chaperone protein ClpB1 | chaperones, ClpB |
| EVM0009750 | AT1G01470 | LEA 14 | Desiccation protectant protein Lea14 | chaperones, LEA14, protectant protein |
| EVM0020529 | AT1G07400 | HSP17.8 | 17.8 kDa class I heat shock protein | chaperones, protectant protein |
| EVM0010334 | AT5G06760 | LEA46 | Late embryogenesis abundant protein 46 | chaperones, protectant protein |
| EVM0013157 | AT4G10250 | HSP20 | 22.0 kDa heat shock protein | chaperones, protectant protein |
| EVM0020522 | AT2G32120 | HSP70-8 | Heat shock 70 kDa protein 8 | chaperones, protectant protein |
| EVM0000970 | AT1G07400 | HSP17.8 | 17.8 kDa class I heat shock protein | chaperones, protectant protein |
| EVM0009127 | AT1G54050 | HSP17.4 | 17.4 kDa class III heat shock protein | chaperones, protectant protein |
| EVM0005203 | AT1G07400 | HSP17.8 | 17.8 kDa class I heat shock protein | chaperones, protectant protein |
| EVM0005043 | AT1G09080 | HSP70 | Heat shock 70 kDa protein | chaperones, protectant protein |
| EVM0013558 | AT5G12020 | HSP17.6 | 17.6 kDa class II heat shock protein | chaperones, protectant protein |
| EVM0013597 | AT1G07400 | HSP17.8 | 17.8 kDa class I heat shock protein | chaperones, protectant protein |
| EVM0015025 | AT2G37770 | AKRC9 | NADPH-dependent aldo-keto reductase, chloroplastic | detoxification |
| EVM0005142 | AT2G34360 | DTX15 | Protein DETOXIFICATION 16 | detoxification |
| EVM0019947 | AT2G30360 | CIPK11 | CBL-interacting serine/threonine-protein kinase 11 | signal transduction |
| EVM0002690 | AT5G36890 | BGLU42 | Beta-glucosidase 42 | signal pathway (ABA signalling) |
| EVM0011202 | AT3G63060 | EDL3 | EID1-like F-box protein 3 | signal pathway (ABA signalling) |
| EVM0017560 | AT1G30100 | NCED1 | 9-cis-epoxycarotenoid dioxygenase NCED1 | signal pathway (ABA signalling) |
| EVM0009136 | AT3G19270 | CYP707A4 | Abscisic acid 8'-hydroxylase 4 | signal pathway (ABA signalling) |
| EVM0007636 | AT3G20015 | ASPG2 | Protein ASPARTIC PROTEASE IN GUARD CELL 2 | signal transduction |
| EVM0001538 | AT1G17545 | PP2C 16 | Protein phosphatase2C 16 | signal transduction (ABA signalling) |
| EVM0015759 | AT3G11410 | PP2C 37 | Protein phosphatase 2C 37 | signal transduction (ABA signalling) |
| EVM0010581 | AT3G11410 | PP2C 37 | Protein phosphatase 2C 37 | signal transduction (ABA signalling) |
| EVM0002096 | AT1G16220 | PP2C06 | Protein phosphatase 2C 06 | signal transduction (ABA signalling) |
| EVM0007696 | AT5G51760 | PP2C 75 | Probable protein phosphatase 2C 75 | signal transduction (ABA signalling) |
| EVM0018487 | AT1G07430 | PP2C03 | Protein phosphatase 2C 03 | signal transduction (ABA signalling) |
| EVM0021555 | AT2G23770 | LYK4 | LysM domain receptor-like kinase 4 | signal transduction |
| EVM0011048 | AT3G05840 | ASK3 | Shaggy-related protein kinase gamma | signal transduction |
| EVM0019350 | AT4G25470 | DREB1D | Dehydration-responsive element-binding protein 1D | transcription factor (ABA pathway) |
| EVM0012434 | AT5G05410 | DREB2C | Dehydration-responsive element-binding protein 2C | transcription factor (ABA pathway) |
| EVM0011123 | AT1G75490 | DREB2G | Dehydration-responsive element-binding protein 2G | transcription factor (ABA pathway) |
| EVM0001110 | AT5G19790 | ERF1 | Ethylene-responsive transcription factor ERN1 | transcription factor |
| EVM0006206 | AT2G46680 | ATHB7 | Homeobox-leucine zipper protein ATHB-7 | transcription factor |
| EVM0000325 | AT5G54070 | HsfA2 | Heat stress transcription factor A-2 | transcription factor |
| EVM0020420 | AT3G24520 | HsfC1 | Heat stress transcription factor C-1 | transcription factor |
| EVM0005395 | AT4G05100 | MYB102 | Transcription factor MYB102 | transcription factor |
| EVM0012782 | AT3G24310 | MYB305 | MYB-like DNA-binding domain protein | transcription factor |
| EVM0020749 | AT3G24310 | MYB305 | MYB-like DNA-binding domain protein | transcription factor |
| EVM0014986 | AT1G08810 | MYB60 | Transcription factor MYB60 | transcription factor |
| EVM0012451 | AT3G24310 | MYB62 | Transcription factor MYB62 | transcription factor |
| EVM0005421 | AT1G52890 | NAC19 | NAC domain-containing protein 19 | transcription factor |
| EVM0003217 | AT1G61110 | NAC25 | NAC transcription factor 25 | transcription factor |
| EVM0002228 | AT1G69490 | NAC29 | NAC transcription factor 29 | transcription factor |
| EVM0007605 | AT1G76880 | DF1 | Trihelix transcription factor DF1 | transcription factor |
| EVM0019285 | AT3G54320 | WRI1 | Ethylene-responsive transcription factor WRI1 | transcription factor |
| EVM0009293 | AT2G47770 | TSPO | Translocator protein homolog | transporter |
| EVM0015361 | AT1G12110 | NPF6.3 | Protein NRT1/ PTR FAMILY 6.3 | transporter |
| EVM0014532 | AT5G35160 | TMN11 | Transmembrane 9 superfamily member 11 | transporter |
| EVM0012269 | AT2G44290 | LTPG14 | Non-specific lipid transfer protein GPI-anchored 14 | transporter |
| EVM0014772 | AT1G71960 | ABCG25 | ABC transporter G family member 25 | transporter |
| EVM0021328 | AT4G10310 | HKT6 | Cation transporter HKT6 | transporter |
| EVM0000643 | AT1G12110 | NPF6.3 | Protein NRT1/ PTR FAMILY 6.3 | transporter |
| EVM0002649 | AT1G69850 | NPF4.6 | Protein NRT1/ PTR FAMILY 4.6 | transporter |
| EVM0003578 | AT1G59740 | NPF4.3 | Protein NRT1/ PTR FAMILY 4.3 | transporter |
| EVM0020774 | At1g32450 | NPF7.3 | Protein NRT1/ PTR FAMILY 7.3 | transporter |
| EVM0005889 | AT1G28220 | PUP1 | Purine permease 1 | transporter |
| EVM0000477 | AT1G28230 | PUP3 | Purine permease 3 | transporter |
| EVM0001789 | AT1G78560 | BASS1 | Probable sodium/metabolite cotransporter BASS1 | transporter |
| EVM0017718 | AT5G09220 | AAP2 | Amino acid transporter AAP2 | transporter |
| EVM0019023 | AT3G48740 | SWEET11 | Bidirectional sugar transporter SWEET11 | transporter |
| EVM0001840 | AT5G23660 | SWEET12 | Bidirectional sugar transporter SWEET12 | transporter |
| EVM0002202 | AT1G09350 | GOLS1 | Galactinol synthase 1 | Osmolyte |

**Table S17.** Detailed annotation of up-regulated genes under salt treatment.

| *Suriana maritima* | *Arabidopsis thaliana* | Gene name | Gene description | Function |
| --- | --- | --- | --- | --- |
| EVM0019406 | AT4G17970 | ALMT12 | Aluminum-activated malate transporter 12 | ion chanel |
| EVM0021328 | AT4G10310 | HKT1 | Sodium transporter HKT1 | ion chanel |
| EVM0002577 | AT2G18960 | H+-ATPase | plasma membrane ATPase | ion chanel |
| EVM0018968 | AT3G16240 | TIP2 | Aquaporin TIP2-1 | water chanel |
| EVM0017560 | AT1G30100 | NCED1 | 9-cis-epoxycarotenoid dioxygenase NCED1 | ABA biosynthesis pathway |
| EVM0018547 | AT1G24580 | XERICO | Probable E3 ubiquitin-protein ligase XERICO | signal pathway (ABA) |
| EVM0011202 | AT3G63060 | EDL3 | EID1-like F-box protein 3 | signal pathway (ABA) |
| EVM0003360 | AT3G07255 | AFP3 | Ninja-family protein AFP3 | signal transduction |
| EVM0012834 | AT5G35410 | SOS2/CIPK24 | CBL-interacting serine/threonine-protein kinase 24 | signal transduction |
| EVM0021555 | AT2G23770 | LYK4 | LysM domain receptor-like kinase 4 | signal transduction |
| EVM0001538 | AT1G17545 | PP2C16 | Protein phosphatase 2C 16 | signal transduction (ABA pathway) |
| EVM0018487 | AT1G07430 | PP2C03 | Protein phosphatase 2C 3 | signal transduction (ABA pathway) |
| EVM0010581 | AT3G11410 | PP2C37 | Protein phosphatase 2C 37 | signal transduction (ABA pathway) |
| EVM0015759 | AT3G11410 | PP2C37 | Protein phosphatase 2C 37 | signal transduction (ABA pathway) |
| EVM0006206 | AT2G46680 | ATHB-7 | Homeobox-leucine zipper protein ATHB-7 | transcription factor |
| EVM0000325 | AT5G54070 | HsfA2 | Heat stress transcription factor A-2 | transcription factor |
| EVM0020420 | AT3G24520 | HsfC1 | Heat stress transcription factor C-1 | transcription factor |
| EVM0017644 | AT3G23250 | MYB15 | Transcription factor MYB15 | transcription factor |
| EVM0012451 | AT3G24310 | MYB62 | Transcription factor MYB62 | transcription factor |
| EVM0005421 | AT1G52890 | NAC19 | NAC domain-containing protein 19 | transcription factor |
| EVM0009293 | AT2G47770 | TSPO | Translocator protein homolog | transporter |
| EVM0006647 | AT5G06530 | ABCG22 | ABC transporter G family member 22 | transporter |
| EVM0014772 | AT1G71960 | ABCG25 | ABC transporter G family member 25 | transporter |
| EVM0012269 | AT2G44290 | LTPG14 | Non-specific lipid transfer protein GPI-anchored 14 | transporter |
| EVM0006335 | AT5G64080 | LTPG31 | Non-specific lipid transfer protein GPI-anchored 31 | transporter |
| EVM0000643 | AT1G12110 | NPF6.3 | Protein NRT1/ PTR FAMILY 6.3 | transporter |
| EVM0015952 | AT1G12940 | NRT2.5 | High affinity nitrate transporter 2.5 | transporter |
| EVM0001840 | AT5G23660 | SWEET12 | Bidirectional sugar transporter SWEET12 | transporter |
| EVM0009424 | AT2G40170 | EM6 | Em-like protein GEA6 | protectant protein |
| EVM0002202 | AT1G09350 | GOLS1 | Galactinol synthase 1 | protectant protein |
| EVM0010744 | AT3G05620 | PME22 | Putative pectinesterase/pectinesterase inhibitor 22 | cell wall modification |
| EVM0019108 | AT3G43270 | PME22 | Probable pectinesterase/pectinesterase inhibitor 32 | cell wall modification |
| EVM0001423 | AT5G17420 | CESA7 | Cellulose synthase A catalytic subunit 7 | cell wall modification |
| EVM0012935 | AT5G55730 | FLA1 | Fasciclin-like arabinogalactan protein 1 | cell wall organization |
| EVM0021779 | AT5G03170 | FLA11 | Fasciclin-like arabinogalactan protein 11 | cell wall organization |
| EVM0019639 | AT4G28380 | LRX4 | Leucine-rich repeat extensin-like protein 4 | cell wall organization |
