# Supplementary material for: Genome compaction underlies the molecular adaptation of bay cedar (Suriana maritima) to the extreme habitat on the tropical coral islands
Source: Plant Divers. 2025 Jan 6;47(2):337–40. doi: 10.1016/j.pld.2025.01.002 (PMC11962907; doi:10.1016/j.pld.2025.01.002)
Supplement: Multimedia component 2 [file mmc2.docx]

**Genome compaction underlies the molecular adaptation of Bay cedar to the extreme habitat on the tropical coral islands**

Miaomiao Shi, Ping Liang, Zhonglai Luo, Yu Zhang, Shiran Gu, Xiangping Wang, Xin Qian, Shuguang Jian, Kuaifei Xia, Shijin Li, Zhongtao Zhao*, Tieyao Tu*, Dianxiang Zhang

**Supplementary Materials**

**1 Plant material and sequencing**

The Bay cedar individuals about 60 cm in height were collected from Yongxing Island (16°50.1′N, 112°19.8′E) of the Paracel Islands and immediately transplanted to a greenhouse in South China Botanical Garden, Chinese Academy of Sciences. To gain a high-quality genome, we employed a combination of three sequencing strategies: long read Nanopore sequencing, short read Illumina sequencing and high-throughput chromosome conformation capture (Hi-C) approach. Fresh leaves from one healthy individual of Bay cedar were harvested and frozen immediately in liquid nitrogen. The genomic DNA was extracted by a modified CTAB method for both Illumina and Nanopore sequencing . For Illumina sequencing, a pair-end (PE) library with an insert size of 350 bp was constructed and sequenced using an Illumina novaseq6000 platform. To produce Oxford Nanopore long reads, ~15 μg of genomic DNA was used to process for a Nanopore SMRT library according to the manufacture’s standard protocol, which was sequenced on PromethlON DNA sequencer (Oxford Nanopore). Fresh leaves of the same individual of Bay cedar were sampled for Hi-C experiment. In brief, a PE150 Hi-C library was constructed through a series of steps including cell cross-linking, restriction endonuclease digestion, terminal repair, cyclization, DNA purification and capture. Subsequently, the prepared library was then sequenced using an Illumina platform. All of the raw reads were trimmed to remove the adaptors and enhance quality.

**2 Genome assembly and quality assessment**

Genome size and heterozygosity were evaluated by k-mer analysis using Illumina sequence data, and the formula of G = k-mer_number/k-mer_depth was used to estimate genome size of Bay cedar (Liu et al., 2013).

For genome assembly, an initial read correction of clean data from Nanopore sequencing was conducted using CANU v1.5 (Koren et al., 2017). Then the assembly was performed by SMARTdenovo (https://github.com/ruanjue/smartdenovo). The consensus assembly was generated by three rounds of RACON v1.32 (Vaser et al., 2017) and three rounds of PILON v1.21 (Walker et al., 2014) polishing using Nanopore and Illumina reads, respectively. To anchor scaffolds onto chromosomes, the Hi-C clean data was first aligned to the draft genome assembly using BWA v0.7.17 (Li and Durbin, 2009). Valid reads from uniquely mapped read pairs were classified by HiC-Pro v2.11.1 (Servant et al., 2015) and retained for subsequent assembly. After manually correcting the mismatched regions between Hi-C assembly and original assembly, scaffolds were clustered, ordered and directed onto 9 pseudochromosomes with LACHESIS software (Burton et al., 2013).

To assess the accuracy and completeness of the genome assembly, we first aligned the Illumina short reads to the assembly with BWA. Next, CEGMA v2.5 (Parra et al., 2007) was used to detect 458 core eukaryotic genes in the genome. In addition, 1440 conserved Benchmarking Universal Single-copy orthologs (BUSCO) in the BUSCO embryophyte odb9 dataset were searched against the genome using BUSCO v2.0 (Simão et al., 2015) to evaluate the completeness of the genome assembly.

**3 Genome annotations**

Annotation of protein-coding genes was conducted in the Bay cedar genome using a combination of *de novo*-based, homology-based and RNA-sequencing (RNA-Seq)-based predictions. For *de novo* gene prediction, five *de novo* prediction programs were utilized with the Bay cedar gene models, including Genscan (Burge and Karlin, 1997), Augustus v2.4 (Stanke et al., 2006), GlimmerHMM v3.0.4 (Majoros et al., 2004), GenelD v1.4 (Blanco et al., 2007) and SNAP (Korf, 2004). For homology-based prediction, the protein sequences of four different species (*Arabidopsis thaliana*, *Arachis hypogaea*, *Glycine max* and *Oryza sativa*) were downloaded and aligned with the repeat-masked Bay cedar genome using GeMoMa v1.3.1 (Keilwagen et al., 2016). For RNA-Seq-based prediction, all RNA-seq data from fresh leaf samples were mapped to the Bay cedar genome using Hisat v2.0.4 (Kim et al., 2015) and assembled by Stringtie v1.2.3 (Pertea et al., 2015), followed by gene prediction with TransDecoder (http://transdecoder.sourceforge.net/) and GeneMarkS-T v5.1 (Tang et al., 2015). Additionally, a *de novo* transcriptome assembly was performed by Trinity v2.1.1 (Haas et al., 2013), and PASA v2.0.2 (Haas et al., 2003) was then used for gene prediction. Finally, all predictions of gene models yielded from the three approaches were integrated using EVidenceModeler (EVM) v1.1.1 (Haas et al., 2008) to generate a consensus gene set.

Functional annotations of the predicted protein-coding genes were conducted by sequencing similarity using BLAST against publicly available protein databases with an E-value cutoff of 1e-5, including the non-redundant protein database of NCBI (NR), KOG, KEGG, TrEMBL. GO annotation was applied with Blast2GO v4.1 (Conesa et al., 2005). For non-coding RNA prediction, transfer RNAs (tRNAs) were predicted by tRNAscan-SE v1.3.1 (Lowe and Eddy, 1997), while ribosomal RNAs (rRNAs) and microRNAs were identified by Infernal v1.1 (Nawrocki and Eddy, 2013) against the Rfam database v13.0 (Griffithsjones et al., 2005).

The repeat annotation of the Bay cedar genome assembly was performed by first using Repeat Modeller (https://www.repeatmasker.org/RepeatModeler/) for de novo identification of repeats and generation of their consensus sequences, which were then used as a customized database for annotating repeats using RepeatMasker (https://www.repeatmasker.org/RepeatMasker/). To further improve LTR annotation, the genome sequences were processed with LTR_finder (Xu and Wang, 2007), which provides the positions of the terminal repeats and internal coding sequences for full-length LTRs. The results were then integrated with the RepeatMasker output to generate a final LTR annotation. The composition of TEs including the ratio of solo-LTRs out of the total LTR insertions was obtained. A set of customized Perl scripts were used in the above process beyond the listed tools. For comparison, the same processing was applied to five other published genomes, including two Fabales species growing in non-extreme habitats: *Dalbergia odorifera* (Hong et al., 2020) and *Polygala tenuifolia* (Meng et al., 2023), and three species living in stressful environments: *Avicennia marina* (Natarajan et al., 2021), *Kandelia obovate* (Hu et al., 2020) and *Rhizophora apiculata* (Xu et al., 2017).

**4 Gene family analysis and phylogenetic tree reconstruction**

Protein-coding gene sequences from eight species of angiosperms were downloaded from Phytozome, NCBI or specific species websites (seen Table S1) to examine evolution and divergence of the Bay cedar genome. Considering the lack of genomic information for closely related species within Surianaceae, we selected six species from the close family Fabaceae, including *Aeschynomene evenia*, *Amphicarpaea edgeworthii*, *Lotus japonicus*, *Phaseolus vulgaris*, *Senna tora*, and *Trifolium pratens*, and two species (*Arabidopsis thaliana* and *Prunus mume*) as outgroups. We only selected the longest transcript of genes for further analysis. Orthofinder v2.4.0 (Emms and Kelly, 2015) was used to infer orthologous groups from Bay cedar and the eight species.

Protein sequences from 1,679 single-copy orthologous genes were used for phylogenetic tree construction. We aligned the protein sequences by MUSCLE v3.8.425 (Edgar, 2004) with default parameters. Based on these alignments, Bayesian Evolutionary Analysis Sampling Trees (BEAST) v2.6.0 (Drummond et al., 2012) was used to construct a phylogeny of the nine species and estimate species divergence times using a Bayesian relaxed molecular-clock method. Two calibration points were set according to Zhao et al. (2021): a split between Fabids and Arabidopsis (mean: 108 Mya, Std dev: 2 Mya), and the stem age of Legume clade (mean: 67.3 Mya, Std dev: 2 Mya). We ran the molecular clock analyses for 100,000,000 generations with sampling every 1,000 states, and ensuring that all parameters reached convergence and effective sample size larger than 200.

**5 Expansion and contraction of gene families**

To examine the expansion and contraction of gene families, the Computational Analysis of gene Family Evolution (CAFÉ) program v4.2.1 (Han et al., 2013) was used to compute changes in gene families along each lineage of the phylogenetic tree under a random birth-and-death model. The gene counts per gene family for each species from Orthofinder were used. Before performing CAFÉ, we filtered the orthologous groups to remove gene families with their members only appearing in one species and gene counts greater than 100 in one or more species. Using conditional likelihoods, the corresponding p values for each lineage were calculated and *P* < 0.01 was set as a threshold to identify rapidly evolved gene families that had undergone significant expansion or contraction. Subsequently, the expanded and contracted gene families in Bay cedar were analyzed for GO enrichment using topGO-package (Alexa et al., 2006) with R by setting all the Bay cedar genes as background.

**6 Genome synteny and whole-genome duplication**

To evaluate the degree of collinearity, we identified syntenic blocks both within and across genomes using the python version MCScan (Tang et al., 2008), which is implemented in JCVI (https://github.com/tanghaibao/jcvi). Only blocks with a minimum of 30 shared genes were retained. Additionally, the WGD events were analyzed for Bay cedar and three Legume species (*A. evenia*, *L. japonicus* and *P. vulgaris*) based on *K*s method (the number of synonymous substitutions per synonymous site). First, we detected putative homologous genes according to BLASTP with an E-value cut-off of 1e-5 by searching the protein sequences within and between species against one another to detect. Then, based on the homologous genes, the WGDI package (Sun et al., 2022) was used for identifying collinear anchor pairs, calculating *K*s values of each anchor gene pair located in syntenic blocks and plotting the *K*s distributions under Gaussian mixture models with the *K*s median value. We eliminated *K*s values that were less than 0.05 to minimize the effects of short tandem repeats. Assuming a constant rate of accumulation for synonymous mutations, we estimated *r* value (average *K*s/year rate) in Fabales by analyzing the *K*s distributions of paralogous genes using the formula: *T* (divergence time) = *K*s/2*r*. The ages of WGD events were then estimated by applying the average *K*s/year rate.

**7 Identification of genes related to stress tolerance**

We collected manually gene families associated with the regulation of abiotic stress tolerance from published papers of *A. thaliana* and *Oryza sativa*, and extracted the protein sequences in *A. thaliana* and *O. sativa*. All genes of the Bay cedar genome were first searched against the sequences of *A. thaliana* and *O. sativa* by BLASTP with an E-value cutoff of 1e-10. Then we submitted the candidate genes to Conserved Domain Database (https://www.ncbi.nlm.nih.gov/cdd) and PfamScan (https://www.ebi.ac.uk/Tools/pfa/pfamscan/) to confirm the presence of domains. In addition, transcription factors were identified for the genome of Bay cedar using iTAK (Zheng et al., 2016) and classified them into categories according to PlantTFDB (http://planttfdb.gao-lab.org/) (Jin et al., 2017).

**8 Transcriptome sequencing and differential expression analysis**

To examine genome-wide responses to salt and drought stresses of Bay cedar, the transcriptome analysis was performed with individuals about 40 cm high, which were transplanted from Yongxing Island of Paracel Islands to South China Botanical Garden, Chinese Academy of Sciences, Guangzhou. The substrate of each pot had the same weight, containing a mixture of coral sand, red loam, coconut bran, peat and organic fertilizer, with a ratio of 8:1:0.5:0.3:0.2. The plants were divided into five treatment groups, which include (i) control (C), irrigated with water; (ii) salt stress (S), treated under 800 mM NaCl for 72 h; (iii) drought stress (D), irrigated nothing until sampling when leaves became wilted; (iv) rewatering for 72 h after salt stress treatments (W_S_) and (v) drought stress treatments (W_D_). Roots were collected for each treatment with three independent biological replicates. Total RNA was extracted with HiPure Universal RNA Mini Kit (Magen, Guangzhou, China). Then Illumina sequencing was conducted for RNA to generate paired-end reads with the length of 150 bp. FASTP (Chen et al., 2018) was used to filter the raw sequencing reads. Then, the clean reads were mapped to the Bay cedar genome using HISAT2 (Kim et al., 2019). FEATURECOUNTS (Liao et al., 2014) incorporated in SUBREAD package (Liao et al., 2013) was applied to estimate gene expression levels. We used the R package DESEQ2 v1.32.0 (Love et al., 2014) to conduct differential expression analysis. Genes with at least twofold changes in expression (false discovery rate, FDR < 0.05) were assigned as differentially expressed genes (DEGs). Key gene expression profiles were visualized using TBtools (Chen et al., 2020) with z-score normalization in the row scale.

**9 Positive selection analysis**

Positive selection analysis was performed for single-copy orthologous genes and the upregulated DEGs. We first identified single-copy orthologous and the high-quality orthologs of the upregulated DEGs from Bay cedar and five Fabaceae species (*A. evenia*, *A. edgeworthii*, *L. japonicus*, *P. vulgaris* and *T. pratense*). Then, based on the phylogenetic topology, the optimized branch-site model incorporated in the PAML package (Yang, 2007) was used to detect positively selected genes (PSGs). We set the branch of Bay cedar as a foreground branch and that of Fagaceae as the background branch. The likelihood ratio tests were conducted between the alternative and null models to determine whether the foreground branch might undergo positive selection. The genes were considered as PSGs when *P* values < 0.05.

**References**

Alexa, A., Rahnenführer, J., Lengauer, T., 2006. Improved scoring of functional groups from gene expression data by decorrelating GO graph structure. Bioinformatics 22, 1600-1607.

Blanco, E., Parra, G., Guigó, R., 2007. Using geneid to identify genes. Curr. Protoc. Bioinf. 18, 4.3.1-4.3.28.

Burge, C., Karlin, S., 1997. Prediction of complete gene structures in human genomic DNA. J. Mol. Biol. 268, 78-94.

Burton, J.N., Adey, A., Patwardhan, R.P., et al., 2013. Chromosome-scale scaffolding of de novo genome assemblies based on chromatin interactions. Nat. Biotechnol. 31, 1119-1125.

Chen, C., Chen, H., Zhang, Y., et al., 2020. TBtools: an integrative toolkit developed for interactive analyses of big biological data. Mol. Plant 13, 1194-1202.

Chen, S., Zhou, Y., Chen, Y., et al., 2018. FASTP: an ultra-fast all-in-one FASTQ preprocessor. Bioinformatics 34, 884–890.

Conesa, A., Götz, S., García-Gómez, J.M., et al., 2005. Blast2GO: A universal tool for annotation, visualization and analysis in functional genomics research. Bioinformatics 21, 3674–3676.

Drummond, A.J., Suchard, M.A., Xie, D., et al., 2012. Bayesian phylogenetics with BEAUti and the BEAST 1.7. Mol. Biol. Evol. 29, 1969-1973.

Edgar, R.C., 2004. MUSCLE: multiple sequence alignment with high accuracy and high throughput. Nucleic Acids Res. 32, 1792–1797.

Emms, D.M., Kelly, S., 2015. OrthoFinder: solving fundamental biases in whole genome comparisons dramatically improves orthogroup inference accuracy. Genome Biol. 16, 157.

Griffithsjones, S., Moxon, S., Marshall, M., et al., 2005. Rfam: annotating non-coding RNAs in complete genomes. Nucleic Acids Res. 33, D121.

Haas, B.J., Delcher, A.L., Mount, S.M., et al., 2003. Improving the *Arabidopsis* genome annotation using maximal transcript alignment assemblies. Nucleic Acids Res. 31, 5654–5666.

Haas, B.J., Papanicolaou, A., Yassour, M., et al., 2013. De novo transcript sequence reconstruction from RNA-seq using the Trinity platform for reference generation and analysis. Nat. Protoc. 8, 1494–1512.

Haas, B.J., Salzberg, S.L., Zhu, W., et al., 2008. Automated eukaryotic gene structure annotation using EVidenceModeler and the Program to Assemble Spliced Alignments. Genome Biol. 9, R7.

Hong, Z., Li, J., Liu, X., et al., 2020. The chromosome-level draft genome of *Dalbergia odorifera*. GigaScience 9, 1-8.

Hu, M.-J., Sun, W.-H., Tsai, W.-C., et al., 2020. Chromosome-scale assembly of the *Kandelia obovata* genome. Hortic. Res. 7, 75.

Jin, J., Tian, F., Yang, D.C., et al., 2017. PlantTFDB 4.0: toward a central hub for transcription factors and regulatory interactions in plants. Nucleic Acids Res. 45, D1040-D1045.

Keilwagen, J., Wenk, M., Erickson, J.L., et al., 2016. Using intron position conservation for homology-based gene prediction. Nucleic Acids Res. 44, e89.

Kim, D., Langmead, B., Salzberg, S.L., 2015. HISAT: A fast spliced aligner with low memory requirements. Nat. Methods 12, 357–360.

Kim, D., Paggi, J., Park, C., et al., 2019. Graph-based genome alignment and genotyping with HISAT2 and HISAT-genotype. Nat. Biotechnology 37, 907–915.

Koren, S., Walenz, B.P., Berlin, K., et al., 2017. Canu: scalable and accurate long-read assembly via adaptive k-mer weighting and repeat separation. Genome Res. 27, 722-736.

Korf, I., 2004. Gene finding in novel genomes. BMC Bioinf. 5, 59.

Li, H., Durbin, R., 2009. Fast and accurate short read alignment with Burrows-Wheeler transform. Bioinformatics 25, 1754-1760.

Liao, Y., Smyth, G., Shi, W., 2013. The SUBREAD aligner: fast, accurate and scalable read mapping by seed-and-vote. Nucleic Acids Res. 41, e108.

Liao, Y., Smyth, G., Shi, W., 2014. FEATURECOUNTS: an efficient general purpose program for assigning sequence reads to genomic features. Bioinformatics 30, 923–930.

Liu, B., Shi, Y., Yuan, J., et al., 2013. Estimation of genomic characteristics by analyzing k-mer frequency in de novo genome projects. arXiv: Genomics 1308.2012v1302.

Love, M., Huber, W., Anders, S., 2014. Moderated estimation of fold change and dispersion for RNA-seq data with DESEQ2. Genome Biol. 15, 550.

Lowe, T.M., Eddy, S.R., 1997. tRNAscan-SE: A program for improved detection of transfer RNA genes in genomic sequence. Nucleic Acids Res. 25, 955–964.

Majoros, W.H., Pertea, M., Salzberg, S.L., 2004. TigrScan and GlimmerHMM: two open source ab initio eukaryotic gene-finders. Bioinformatics 20, 2878–2879.

Meng, F., Chu, T., Feng, P., et al., 2023. Genome assembly of *Polygala tenuifolia* provides insights into its karyotype evolution and triterpenoid saponin biosynthesis. Hortic. Res. 10, uhad139.

Natarajan, P., Murugesan, A.K., Govindan, G., et al., 2021. A reference-grade genome identifies salt-tolerance genes from the salt-secreting mangrove species *Avicennia marina*. Commun. Biol. 4, 851.

Nawrocki, E.P., Eddy, S.R., 2013. Infernal 1.1: 100-fold faster RNA homology searches. Bioinformatics 29, 2933–2935.

Parra, G., Bradnam, K., Korf, I., 2007. CEGMA: a pipeline to accurately annotate core genes in eukaryotic genomes. Bioinformatics 23, 1061-1067.

Pertea, M., Pertea, G.M., Antonescu, C.M., et al., 2015. StringTie enables improved reconstruction of a transcriptome from RNA-seq reads. Nat. Biotechnol. 33, 290–295.

Servant, N., Varoquaux, N., Lajoie, B.R., et al., 2015. HiC-Pro: an optimized and flexible pipeline for Hi-C data processing. Genome Biol. 16, 259.

Simão, F.A., Waterhouse, R.M., Ioannidis, P., et al., 2015. BUSCO: assessing genome assembly and annotation completeness with single-copy orthologs. Bioinformatics 31, 3210-3212.

Stanke, M., Schoffmann, O., Morgenstern, B., et al., 2006. Gene prediction in eukaryotes with a generalized hidden Markov model that uses hints from external sources. BMC Bioinf. 7, 62.

Sun, P., Jiao, B., Yang, Y., et al., 2022. WGDI: A user-friendly toolkit for evolutionary analyses of whole-genome duplications and ancestral karyotypes. Mol. Plant 15, 1841-1851.

Tang, H., Bowers, J.E., Wang, X., et al., 2008. Synteny and collinearity in plant genomes. Science 320, 486–488.

Tang, S., Lomsadze, A., Borodovsky, M., 2015. Identification of protein coding regions in RNA transcripts. Nucleic Acids Res. 43, e78.

Vaser, R., Sovic, I., Nagarajan, N., et al., 2017. Fast and accurate de novo genome assembly from long uncorrected reads. Genome Res. 27, 737-746.

Walker, B.J., Abeel, T., Shea, T., et al., 2014. Pilon: an integrated tool for comprehensive microbial variant detection and genome assembly improvement. PLoS One 9, e112963.

Xu, S., He, Z., Zhang, Z., et al., 2017. The origin, diversification and adaptation of a major mangrove clade (Rhizophoreae) revealed by whole-genome sequencing. Natl. Sci. Rev. 4, 721-734.

Xu, Z., Wang, H., 2007. LTR_FINDER: an efficient tool for the prediction of full-length LTR retrotransposons. Nucleic Acids Res. 35, W265-W268.

Yang, Z., 2007. PAML 4: phylogenetic analysis by maximum likelihood. Mol. Biol. Evol. 24, 1586-1591.

Zhao, Y., Zhang, R., Jiang, K.W., et al., 2021. Nuclear phylotranscriptomics and phylogenomics support numerous polyploidization events and hypotheses for the evolution of rhizobial nitrogen-fixing symbiosis in Fabaceae. Mol. Plant 14, 748-773.

Zheng, Y., Jiao, C., Sun, H., et al., 2016. iTAK: a program for genome-wide prediction and classification of plant transcription factors, transcriptional regulators, and protein kinases. Mol. Plant 9, 1667-1670.
